# Supplementary material for: Melatonin Signaling Controls Circadian Swimming Behavior in Marine Zooplankton
Source: Cell. 2014 Sep 25;159(1):46–57. doi: 10.1016/j.cell.2014.07.042 (PMC4182423; doi:10.1016/j.cell.2014.07.042)
Supplement: Document S1. Article plus Supplemental Information [file mmc2.pdf]

Cell

Melatonin Signaling Controls Circadian Swimming Behavior in Marine Zooplankton

Maria Antonietta Tosches,[1,*] Daniel Bucher,[1,2] Pavel Vopalensky,[1] and Detlev Arendt[1,2,*]
[1]European Molecular Biology Laboratory, Developmental Biology Unit, Meyerhofstrasse 1, 69117 Heidelberg, Germany
[2]Centre for Organismal Studies (COS), University of Heidelberg, 69120 Heidelberg, Germany
*Correspondence: tosches@embl.de (M.A.T.), arendt@embl.de (D.A.)
http://dx.doi.org/10.1016/j.cell.2014.07.042
This is an open access article under the CC BY license (http://creativecommons.org/licenses/by/3.0/).

SUMMARY

Melatonin, the "hormone of darkness," is a key regulator of vertebrate circadian physiology and behavior. Despite its ubiquitous presence in Metazoa, the function of melatonin signaling outside vertebrates is poorly understood. Here, we investigate the effect of melatonin signaling on circadian swimming behavior in a zooplankton model, the marine annelid *Platynereis dumerilii.* We find that melatonin is produced in brain photoreceptors with a vertebrate-type opsin-based phototransduction cascade and a light-entrained clock. Melatonin released at night induces rhythmic burst firing of cholinergic neurons that innervate locomotor-ciliated cells. This establishes a nocturnal behavioral state by modulating the length and the frequency of ciliary arrests. Based on our findings, we propose that melatonin signaling plays a role in the circadian control of ciliary swimming to adjust the vertical position of zooplankton in response to ambient light.

INTRODUCTION

In vertebrates, melatonin and its signaling through G-protein-coupled receptors (GPCRs) play a fundamental role in the circadian modulation of physiology and behavior. Melatonin is a highly diffusible hormone secreted at night by the pineal organ, which in nonmammalian vertebrates is directly light sensitive (Lamb, 2013). As a "hormone of darkness," melatonin levels change according to circadian, lunar, and seasonal cycles (Reiter, 1993). A conserved function of melatonin in vertebrates, from fish to mammals, is the regulation of sleep (Dollins et al., 1994; Zhdanova et al., 2001). In mammals, this occurs through direct modulation of neuronal excitability in the suprachiasmatic nucleus, the brain circadian pacemaker (Jiang et al., 1995), and in the thalamus, where sleep is initiated (Ochoa-Sanchez et al., 2011).

Melatonin is one of the oldest biologically active molecules in nature, present in a nearly ubiquitous range of organisms, including bacteria and plants. Its rhythmic production has been reported not only in vertebrates, but also in various protostomes, in cnidarians, and in dinoflagellates (Balzer and Hardeland, 1991; Hardeland and Poeggeler, 2003; Roopin and Levy, 2012). The ubiquitous presence of melatonin (Hardeland and Poeggeler, 2003) has been linked to its chemical properties, which make it one of the most powerful radical scavengers known in nature (Tan et al., 2007). Beyond that, the presence of melatonin receptors in all animal lineages, except sponges (Feuda et al., 2012), indicates that melatonin acquired an additional signaling function early in animal evolution.

It has been speculated that the role of melatonin as the "hormone of darkness" has evolved directly from its antioxidant properties: in a system with constant melatonin synthesis, the reduction of melatonin levels during the day due to its light-dependent oxidation would make it a suitable signal for darkness (Hardeland et al., 1995). The newly evolving melatonin receptors might thus have recognized melatonin as a "chemical indicator of darkness" for the circadian regulation of some physiological process and/or behavior. However, the nature of such melatonin-controlled behavior has so far remained elusive, as the role of melatonin signaling has been poorly investigated outside vertebrates. In few cases, it has been demonstrated that melatonin has modulatory (often inhibitory) effects on locomotion (Anctil et al., 1991; Bentkowski et al., 2010; Tanaka et al., 2007; Tilden et al., 2003); yet it is not clear whether these effects are linked to circadian rhythms and how they relate to the ancient role of melatonin signaling in animals.

To broaden our perspective on the function of melatonin signaling in metazoans, we investigated its possible role in the day/night control of zooplankton locomotion. Primary larvae forming most of the zooplankton are part of the life cycle in the majority of animal phyla. They swim using locomotor cilia, which are either distributed over the entire body surface or concentrated in specialized ciliary bands. In the ocean, almost all of these larvae show a pronounced rhythmic behavior, known as diel vertical migration (DVM), which generally consists of an upward swimming phase at dusk and a downward displacement phase throughout the night and/or at dawn (Alldredge and King, 1980; Forward, 1988; Rudjakov, 1970). The control of vertical migration in the oceans has been linked to the origin of animal circadian rhythms (Gehring and Rosbash, 2003; Pittendrigh, 1993), as a mechanism to escape damaging UV irradiation during the daytime (Calkins and Thordardottir, 1980; Rhode et al., 2001). We thus considered it an attractive hypothesis that

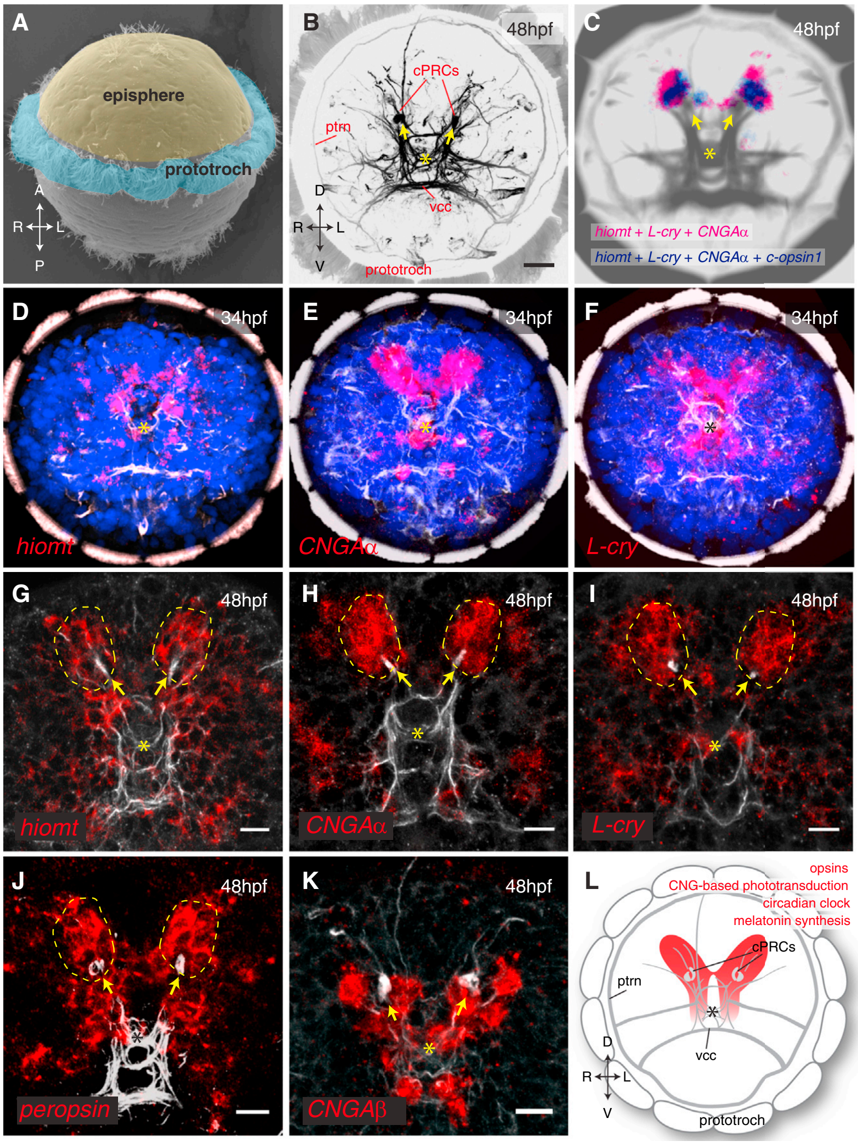

Figure 1. Coexpression of Opsins, Clock Genes, and *hiomt* in the Dorsal Larval Brain

(A) SEM of a *Platynereis* trochophore larva, showing the position of the developing apical nervous system (episphere) and the prototroch (image courtesy of H. Hausen).

(B) Z-projection of the brain axonal scaffold at 48 hpf, stained with an anti-acetylated tubulin antibody, apical view. Main landmarks are indicated. cPRCs, ciliary photoreceptor cells (yellow arrows); ptrn, prototroch ring nerve; vcc, ventral cerebral commissure. Scale bar, 20 μm.

(C) Average gene coexpression at 48 hpf obtained with Profiling by Image Registration (PrImR). Blue, coexpression of *hiomt*, *L-cry*, *CNGAα*, and *c-opsin1*; magenta, coexpression of *hiomt*, *L-cry*, and *CNGAα*; gray, average axonal scaffold.

(D–F) Expression of *hiomt* (D), *CNGAα* (E), and *L-cry* (F) at 34 hpf. All images are Z-projections of the entire episphere (apical views, dorsal up). Blue, DAPI; red, gene expression; gray, axonal scaffold.

(G–K) Expression of *hiomt* (G), *CNGAα* (H), *L-cry* (I), *peropsin* (J), and *CNGAβ* (K) at 48 hpf. All images are 10 μm Z-projections of the extended cPRCs region (apical views, dorsal up). Red, gene expression; gray, axonal scaffold. Arrows, cilia of cPRCs; asterisks, apical organ. Scale bar, 10 μm.

(L) Schematic representation of the larval brain; the region harboring expression of opsins, CNG-based phototransduction, clock genes, and melatonin synthesis markers is highlighted.

See also Figures S1 and S2.

melatonin signaling may play a role in the diurnal control of ciliary swimming. To elucidate the possible interplay between ambient light detection, melatonin signaling, and circadian larval swimming activity in an invertebrate zooplankton larva, we chose the annelid *Platynereis dumerilii*, a marine animal model amenable to molecular and behavioral studies, which has conserved vertebrate-type ciliary photoreceptors (Arendt et al., 2004; Jékely et al., 2008).

RESULTS

Markers of Melatonin Synthesis, Phototransduction, and Circadian Clock Are Coexpressed in the Dorsal Brain of *Platynereis* Larvae

As an entry point, we found that the most specific marker of melatonin synthesis, the gene *hydroxyindole-O-methyltransferase* (*hiomt*) (Nowak et al., 1993), is conserved in several bilaterian genomes (Figure S1 available online). Therefore, in order to identify the *Platynereis* melatonin-producing cells, we performed whole-mount in situ hybridization (WMISH) with probes detecting transcripts of *Platynereis hiomt* and of marker genes for phototransduction and circadian clock (Figure 1). Expression was detected in the episphere, where the larval brain differentiates (Figures 1A and 1B), starting at 34 hr postfertilization (hpf) in dorsomedial cells (Figures 1D–1F) and covering the extended ciliary photoreceptor region at 48 hpf (dashed circles in Figures 1G–1J). This area comprises the previously described ciliary photoreceptors expressing *c-opsin1* (Arendt et al., 2004). In vertebrate ciliary photoreceptors, phototransduction involves the G-alpha subunit G*t*, a member of the G*i* family, and nonselective cationic cyclic nucleotide-gated (CNG) channels, which lead to an OFF response (hyperpolarization) in the presence of light. The same phototransduction components were expressed specifically in the *Platynereis* dorsal brain (Figures S2A–S2C, 1E, 1H, and 1K). Intriguingly, markers of the circadian clock, like *bmal* (Arendt et al., 2004), *vrille*, and *L-cry*, the ortholog of the *Drosophila* light-sensitive cryptochrome, were expressed in the same region (Figures S2D, 1F, and 1I; see also Zantke et al., 2013). Coexpression of these genes was apparent from the similar position of stained cells adjacent to the photoreceptor cilia (arrows in Figure 1) and

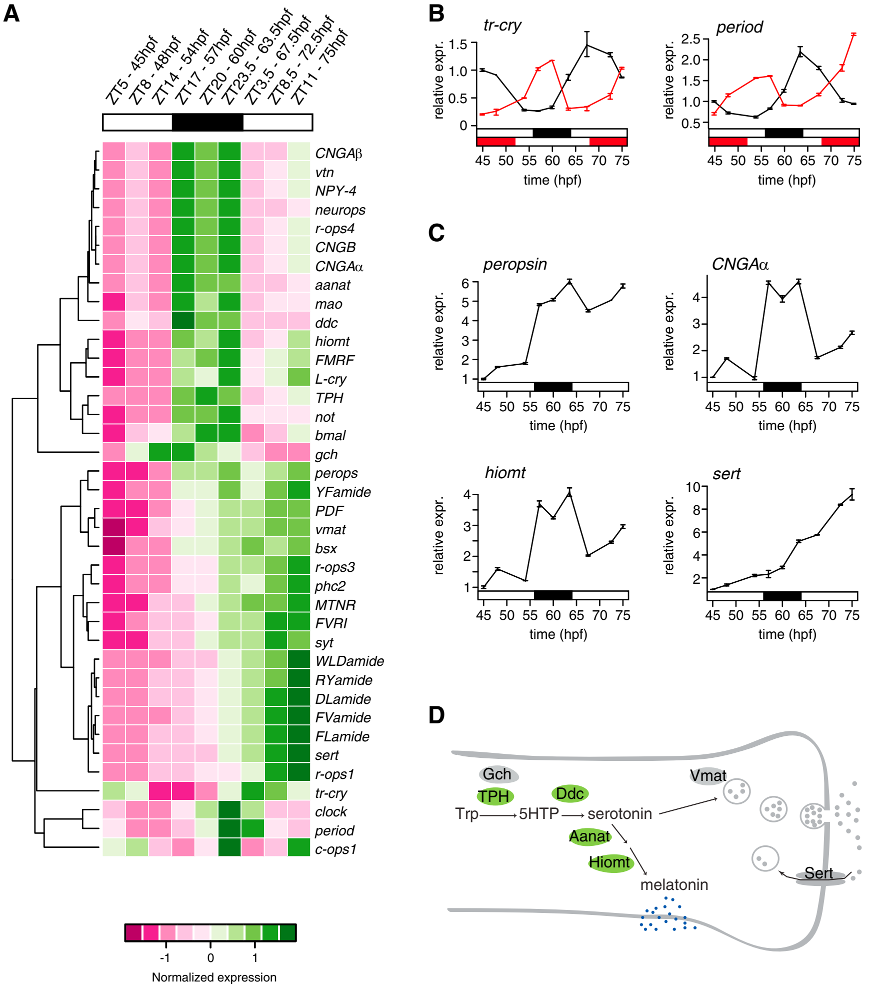

Figure 2. Global Changes of Gene Expression during the Light-Dark Cycle

(A–D) Gene expression profiling of *Platynereis* larvae between 45 and 75 hpf with Fluidigm Dynamic Arrays. (A) Hierarchical clustering of scaled gene expression levels. Sampling stages and circadian times indicated at the top. High and low normalized expression levels are indicated with green and magenta, respectively. (B and C) Expression profiles from the Fluidigm screen. Gene expression levels are plotted as relative to the expression at 45 hpf. Each point is the average of the two technical replicates (see the Extended Experimental Procedures for details). Error bars indicate SD. (B) Expression of the clock genes *tr-cry* and *period* between 45 and 75 hpf. The red lines indicate relative expression in siblings raised under an inverted light-dark cycle (the red solid bars below indicate the night phase of the inverted cycle). (C) Examples of day-night gene expression dynamics. *CNGAα* and *hiomt* are upregulated at night; *sert* is developmentally regulated; and *peropsin* is both night upregulated and developmentally regulated. (D) Schematic of the serotonin-melatonin biosynthesis pathway. The genes significantly upregulated at night are shown in green. Hpf, hours post fertilization; ZT, *zeitgeber* time.

See also Figure S3.

confirmed with the Profiling by Image Registration (PrImR) technique (Tomer et al., 2010), which revealed coexpression of *Platynereis hiomt*, *L-cry*, *CNGAα*, and *c-opsin1* in a few cells of the dorsal brain, including the ciliary photoreceptors (blue in Figure 1C). Interestingly, the expression of melatonin synthesis, phototransduction, and clock markers was broader than the highly restricted *c-opsin1* expression (magenta in Figure 1C) and overlapped with that of other opsin family representatives, such as *neuropsin* (Figure S2E) and *peropsin* (Figure 1J; compare extended red coexpression region in summary Figure 1L). Therefore, the *Platynereis* dorsal brain is a complex site of nonvisual light sensitivity. Our data suggest that photosensitivity, circadian entrainment, and melatonin release are directly coupled, at the cellular level, in the *Platynereis* dorsal brain, just as in pineal photoreceptors.

Melatonin-Synthesis and Phototransduction Genes Are Specifically Upregulated at Night

The first experimental evidence for such coupling was obtained from a quantification of gene expression dynamics during the light-dark cycle. We measured simultaneously the expression levels of clock genes, neuropeptides, opsins, and genes of the melatonin synthesis pathway using a high-throughput quantitative PCR (qPCR) screen (Figures 2 and S3). To this aim, *Platynereis* larvae were sampled between 45 and 75 hpf (with the night phase between 56 and 64 hpf). The circadian system is already active and entrainable at these larval stages: the expression levels of clock genes (such as *clock*, *period*, and *tr-cry*; Zantke et al., 2013) oscillated throughout the light-dark cycle, and in sibling larvae raised in an inverted light-dark cycle (shifted by 12 hr), the phase of clock gene expression was likewise shifted by 12 hr (Figure 2B). Clustering analysis of gene expression changes over our entire data set retrieved two major groups: genes with expression levels constantly increasing throughout development (reflecting the steady increase in the number of differentiated cells at these stages) and genes upregulated specifically during the night (Figures 2A, 2C, and S3). The first group included neuronal differentiation markers, such as *synaptotagmin* (*syt*) and *prohormone convertase 2* (*phc2*), several neuropeptides (Conzelmann et al., 2011), and rhabdomeric opsins expressed in the eyes (*r-ops1* and *r-ops3*; Randel et al., 2013). The second group included several genes expressed in the *hiomt*+ cells, such as *peropsin*, *neuropsin*, and *CNG* channel subunits, indicating a night-dependent change of light sensitivity. Moreover, all genes involved in the melatonin synthesis and degradation pathway (*TPH*, *ddc*, *mao*, *aanat*, and *hiomt*) were upregulated at night, whereas those exclusively involved in serotonin signaling (*vmat*, *sert*) were not (Figures 2C and 2D). These findings suggested that melatonin itself is produced and released during the night, as reported in other invertebrate species (Hardeland and Poeggeler, 2003),

and that melatonin release is directly controlled by light and/or by the circadian clock.

Melatonin Signaling Establishes a Nocturnal Behavioral State

To identify and characterize a possible effect of nocturnal melatonin release on larval behavior, we assayed larval ciliary swimming. In *Platynereis*, the larval brain controls ciliary locomotion by direct innervation of the prototroch, an equatorial girdle of cells equipped with multiple motile cilia (Figures 1A and 1B). Changes in the ciliary beating frequency (CBF) and in ciliary closure time (i.e., length and frequency of ciliary arrests) differentially affect swimming speed, depth, and direction (Conzelmann et al., 2011; Jékely et al., 2008). Thus, we assayed CBF and ciliary arrests at 52 hpf, comparing larvae in their midday and midnight (ZT8 and ZT20; ZT, zeitgeber time; Figure 3A). Although CBF did not differ significantly between day and night (Figure 3B), we found a significant increase in overall ciliary closure time at night (Figure 3C). Treatment at night with the melatonin receptor antagonist luzindole dramatically decreased closure time (Figure 3D), indicating that the nocturnal increase in ciliary closure time depends on melatonin signaling. Consistently, daytime treatment with 100 μM melatonin was sufficient to almost double ciliary closure time (Figure 3E). Finally, we found that absence of light during daytime was not sufficient by itself to increase ciliary closure time (Figure 3F). Our data are thus consistent with the possibility that the circadian clock contributes to the regulation of the melatonin rhythm (as in vertebrates; Falcón et al., 2010).

Next, we reasoned that the ciliary closure time depends on two parameters: the frequency and the length of the closure events. Therefore, we dissected how these parameters change during day and night. At night, the frequency of ciliary arrests is significantly higher compared to daytime (Figure 3G). Moreover, the bimodal distribution of ciliary closure lengths (Figure 3H) indicated the existence of two distinct kinds of arrests, the ‘‘short’’ and ‘‘long’’ closures (with an average length of 0.2 and 2.2 s, respectively; Figure 3H). The day-night transition involves a significant increase of the proportion of long closures (Figures 3I and 3I’). The melatonin receptor antagonist luzindole applied at night recapitulated the diurnal condition: the overall frequency of arrests was reduced (Figure 3J) and the long closures were suppressed (Figures 3K and K′). Conversely, treatment with melatonin during the day increased the proportion of long closures to night levels (Figures 3L and 3L′). Taken together, our data indicate the existence of two alternative behavioral states during day and night, which correlate with the day-night differences in gene expression profile. Melatonin signaling is necessary and sufficient to increase ciliary closure frequency and length, establishing the nocturnal behavioral state.

Cholinergic Neurons Control Ciliary Arrests

To dissect the mechanism of ciliary arrest, we set out to identify the ‘‘ciliomotor neurons’’ driving ciliary arrests and to test whether they are responsive to melatonin signaling. Given that the prototroch cells express the *acetylcholine receptor* α*9/10* (Jékely et al., 2008), the best candidates were the early developing cholinergic neurons previously identified in the larval brain (Jékely et al., 2008). Consistent with this, application of acetylcholine (ACh) significantly increased ciliary closure time, whereas the acetylcholine receptor antagonist mecamylamine was able to suppress the ACh effect and reduce ciliary arrests (Figure 4A). Notably, mecamylamine specifically suppressed the long closures that are regulated by melatonin signaling (Figure 4B). Acetylcholinesterase staining confirmed direct innervation of the prototroch by cholinergic axons (Figure 4C). In particular, the ventral cholinergic neurons, some of which directly innervate the prototroch (cfr MN3l/MN3r in Randel et al., 2014), qualified as candidate melatonin-responsive neurons, as indicated by the coexpression of the *Platynereis melatonin receptor* (*MTNR*; Figure S2) and the cholinergic marker *ChAT* (Figure 4D).

Nocturnal Melatonin Signaling Induces Rhythmic Activity of Cholinergic Ciliomotor Neurons

To test whether melatonin signaling modulates neuronal activity, we performed two-photon imaging on larvae expressing the genetically encoded calcium indicator GCaMP6s. To facilitate the identification of the ventral cholinergic neurons, we took advantage of the fact that these cells are located at the interface between the two halves of the larval brain developing from the AB and CD blastomeres (Figure S4). Therefore, we expressed GCaMP6s and H2B-RFP in the CD lineage by single-blastomere injections (Figure 5A). In control conditions, we detected sparse, arrhythmic small-amplitude calcium transients in these neurons and along their projections. Unexpectedly, the application of melatonin elicited enhanced neuronal activity in the most ventral cell of the cholinergic cluster. This heightened neuronal activity also revealed a direct contralateral projection to the prototroch cells through the ventral cerebral commissure (Figure 5B). Melatonin robustly induced a sustained rhythmic activity pattern, characterized by the appearance of GCaMP6s peaks of regular amplitude and frequency (mean frequency: 0.43 Hz; Figure 5C; Movie S1). The melatonin-induced calcium peaks were readily suppressed by subsequent addition of the melatonin receptor antagonist luzindole (Figure 5C). Moreover, we detected this activity pattern specifically in the prototroch-projecting ciliomotor neuron and never in any of the neighboring CD cells (Figure 5D). To test whether the melatonin-induced activity recapitulates the nocturnal endogenous activity, we performed the same experiments at night. In untreated larvae, we found spontaneous rhythmic calcium peaks reminiscent of the melatonin-induced activity (mean frequency: 0.31 Hz; Figure 5E). These peaks were suppressed by application of luzindole (Figure 5E), indicating that at night this rhythmic activity is induced by endogenous melatonin release. Analysis of GCaMP6s peak frequency and temporal distribution confirmed that daytime administration of melatonin mimics the spontaneous nocturnal activity (Figures 5F and 5G). Notably, the rhythmic activity of the cholinergic neuron proved insensitive to mecamylamine treatment (Figure S5), indicating that the effect of cholinergic agonists and antagonists on ciliary arrests occurs downstream of these neurons (i.e., at the cholinergic ciliomotor synapses on the prototroch cells).

Melatonin-Induced Bursting Enhances Synaptic Transmission

To better understand the neurophysiological processes that underlie the observed rhythmic firing and the nocturnal change in

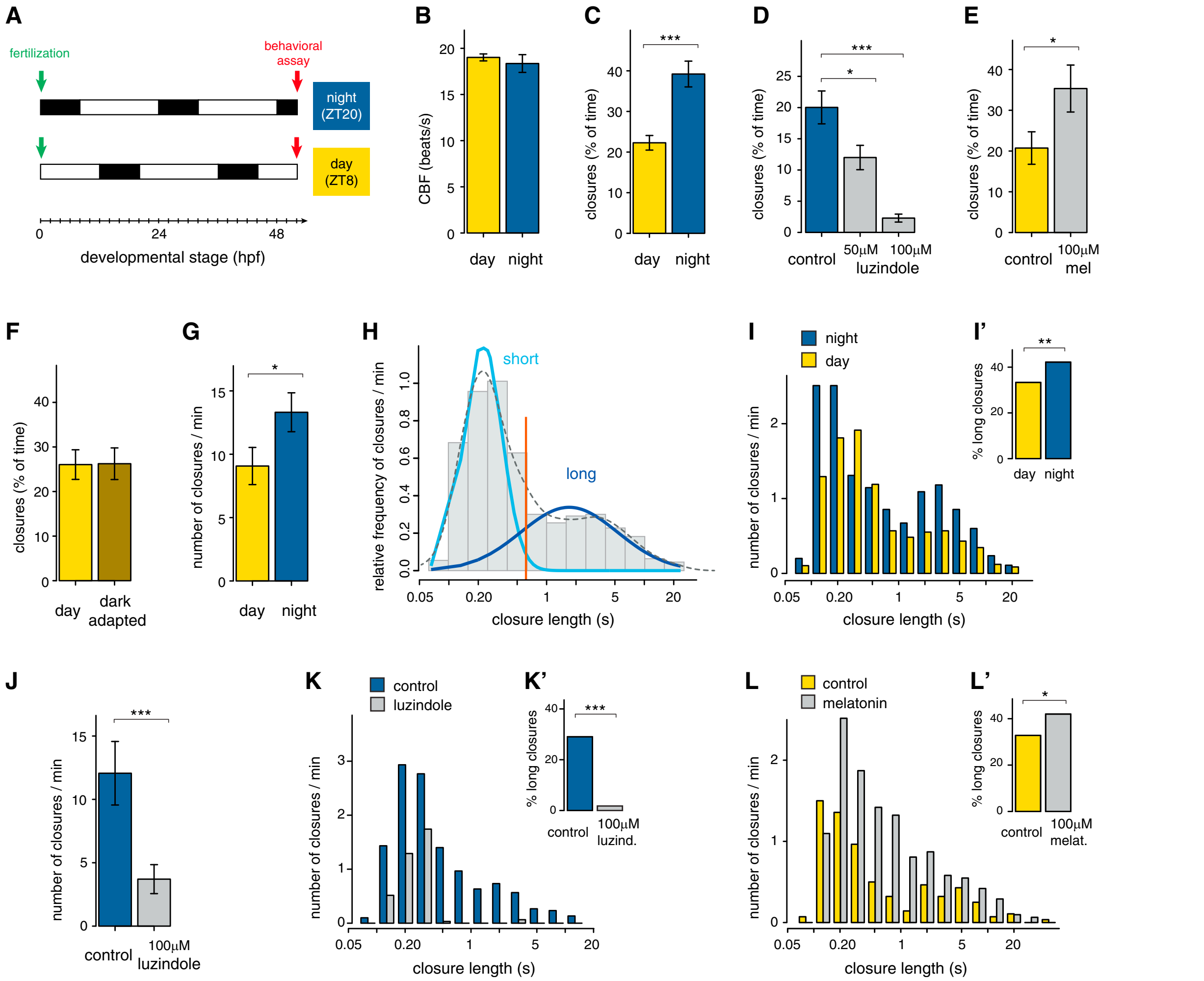

Figure 3. Melatonin Signaling Modulates Nocturnal Locomotor Activity

(A) Design of the behavioral experiments. Larvae were assayed for locomotor activity at 52 hpf (red arrows), corresponding to ZT8 (midday) or ZT20 (midnight).

(B) Ciliary beating frequency (CBF; beats/s) during day and night. n = 59 and 25 larvae. p = 0.44, unpaired t test.

(C) Ciliary closure time (% of total time) during day and night. $p < 0.001$, unpaired t test. n = 72 and 55 larvae.

(D) Ciliary closure time (% of total time) at night, in larvae treated with 50 or 100 μM of the melatonin receptor antagonist luzindole in DMSO (gray). Control = 0.1% DMSO (blue). $p < 0.05$ and $p < 0.001$, unpaired t test. n = 30, 12, and 16 larvae.

(E) Ciliary closure time (% of total time) during daytime; larvae were treated with 100 μM of melatonin in NSW (gray). Control = NSW (yellow). p < 0,05, unpaired t test. n = 28 and 31 larvae.

(F) Ciliary closure time (% of total time) at ZT8; larvae were imaged under normal daylight or in complete darkness after 1 hr of dark adaptation. n = 27 and 23 larvae.

(G) Frequency of ciliary arrests (number of closures/min) during day and night. n = 58 and 55 larvae, respectively. $p < 0.05$, unpaired t test.

(H) Probability density plot of ciliary closure lengths (on the logarithmic scale) during daytime, showing a bimodal distribution with distinct long and short arrests.

(I) Normalized distribution of ciliary closure lengths during day (yellow) and night (blue). n = 58 and 55 larvae.

(I′) Percentage of long ciliary arrests during day and night; data are from (I). Long arrests are 33% and 42% of total arrests, respectively. $p < 0.01$, chi-square test.

(J) Frequency of ciliary arrests (number of closures/min) in controls (0.1% DMSO, blue) and in larvae treated with 100 μM luzindole (gray) during the night. n = 30 and 31 larvae. $p < 0.001$, unpaired t test.

(K) Normalized distribution of ciliary closure lengths in controls (0.1% DMSO, blue) and in larvae treated with 100 μM luzindole (gray). n = 30 and 31 larvae.

(K′) Percentage of long ciliary arrests in controls (0.1% DMSO) and in larvae treated with 100 μM luzindole at night; data are from (K). Long closures are 29% and 2% of total, respectively. $p < 0.001$, chi-square test.

(L) Normalized distribution of ciliary closure lengths in controls (NSW, yellow) and in larvae treated with 100 μM melatonin (gray). n = 24 and 23 larvae.

(L′) Percentage of long ciliary arrests in controls and in larvae treated with 100 μM melatonin during the day; data are from (L). Long closures are 33% and 42% of total, respectively. $p < 0.05$, chi-square test. In (B)–(G) and (J), data are shown as mean ± SEM, and error bars indicate SEM. NSW, natural seawater.

ciliary arrests, we performed intracellular sharp electrode recordings from prototroch cells. Ciliary arrests coincided with electrical activity, as reported previously from extracellular field potential recordings (Conzelmann et al., 2011); however, intracellular recordings with simultaneous imaging of ciliary beating revealed that these arrests coincide with depolarizations that

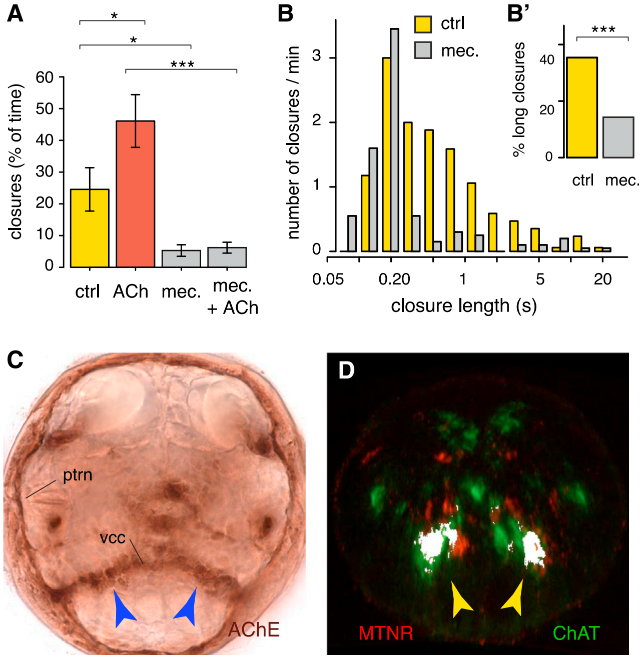

Figure 4. Ciliary Arrests Are Triggered by Cholinergic Transmission

(A) Ciliary closure time (% of total time) during daytime, in controls (NSW, yellow), and in larvae treated with 1 μM ACh (red), with 1 μM of the cholinergic antagonist mecamylamine or with a mixture of 1 μM of mecamylamine and 1 μM ACh (gray). One-way ANOVA with post hoc Holm adjustment, $*p < 0.05$, $***p < 0.001$. n = 15, 19, 22, and 17 larvae, respectively. Error bars represent SEM.

(B) Normalized distribution of ciliary closure lengths in controls (0.95% EtOH, yellow) and in larvae treated with 100 μM mecamylamine (gray). n = 17 and 20 larvae.

(B′) Percentage of long closures in controls (0.95% EtOH) and in larvae treated with 100 μM mecamylamine during the day; data are from (B). Long closures are 35% and 14% of total, respectively. $p < 0.001$, chi-square test.

(C) Acetylcholinesterase (AChE) staining of a 52 hpf *Platynereis* trochophore larva (apical view), showing signal in the prototroch ring nerve (ptrn). The blue arrowheads indicate the ventral brain cholinergic neurons and projections to the ptrn through the ventral cerebral commissure (vcc).

(D) Coexpression (white) of *melatonin receptor* (*MTNR*, red; see also Figure S2) and the cholinergic marker *ChAT* (green) in the 48 hpf larval brain, after image registration. Arrowheads, ventral brain cholinergic neurons.

arise from an intrinsic electrical excitability of the prototroch cell (Figure 6A). Consistent with excitatory cholinergic transmission, application of acetylcholine induced prototroch depolarization, and this effect was suppressed by concurrent mecamylamine application (Figure S6). Application of melatonin caused a significant increase in prototroch spike frequency (Figures 6A–6C), as well as a decrease in the distribution of interspike intervals (Figure 6D), reflecting the sustained electrical activity that would be expected given the effect melatonin has on the length of ciliary arrests.

Intracellular recordings also revealed the presence of excitatory postsynaptic potentials (EPSPs). In agreement with the calcium imaging data, melatonin changed prototroch EPSPs from single sparse events to regular periodic bursts, each characterized by a duration of 0.25 ± 0.08 s (Figure 6E). The effect of melatonin was reversible with application of the melatonin receptor antagonist luzindole (Figure 6E). Moreover, application of mecamylamine after melatonin treatment completely eliminated excitatory synaptic transmission, indicating that this activity was exclusively cholinergic (Figure 6E). These results indicate that each nocturnal peak of GCaMP6s fluorescence corresponds to a burst of action potentials in the presynaptic cholinergic cells, increasing the likelihood of prototroch spiking. To further assess the effect of melatonin-induced bursting on synaptic transmission, we quantified EPSP amplitude before and after application of melatonin. This revealed a significant increase of the mean EPSP amplitude, indicating that the number of synaptic vesicles released per action potential more than doubles during the melatonin-induced bursting (Figures 6F and 6G). At the same time, the mean resting membrane potential of the prototroch cells (which do not express *MTNR*) was not significantly altered in presence of melatonin (from −70.45 to −70.39 mV, n = 7, ns). Therefore, we conclude that at night melatonin signaling directly controls the excitability of presynaptic cholinergic neurons, that it induces rhythmic bursting and potentiates synaptic transmission at the ciliomotor-prototroch cells synapses, and that this augmented release of acetylcholine enhances the frequency and duration of ciliary arrests.

DISCUSSION

Our combination of expression profiling, neuronal activity imaging, and intracellular recordings in a zooplankton model reveals a role of melatonin signaling in the circadian control of ciliary swimming. In *Platynereis*, melatonin is necessary and sufficient to establish a nocturnal behavioral state characterized by enhanced ciliary arrests. We can distill the underlying circuit architecture into two major components (red and blue in Figure 7). The first is the sensory-neuromodulatory component, constituted by melatonin-releasing, ambient light-detecting photoreceptors that harbor a circadian clock. The second is the effector component, represented by the cholinergic ciliomotor neurons, which are direct targets of nocturnal melatonin signaling and respond by rhythmic bursting. This system represents a minimalistic example of integration of sensory-neuromodulatory and motor circuits in animal nervous systems. Comparative evidence indicates that at least parts of these circuits are of more widespread occurrence in animals and may thus be evolutionary ancient.

Complex Integration of Ambient Light in the "Apical Nervous System"

The shared key feature of the dorsal brain neurons described here is the coexpression of *hiomt* with markers of opsin-based phototransduction and the circadian clock. The circadian clock is already entrained in early larvae, and several genes expressed in these cells, including *hiomt* itself, are upregulated at night, suggesting that ambient light detection and the circadian clock control melatonin synthesis. A coupling of these three processes in the same cells is also observed in the pineal photoreceptors of lower vertebrates (Lamb, 2013), and intriguingly, the *Platynereis* ciliary photoreceptors (previously compared to pineal and retinal photoreceptors; Arendt et al., 2004) are part of the

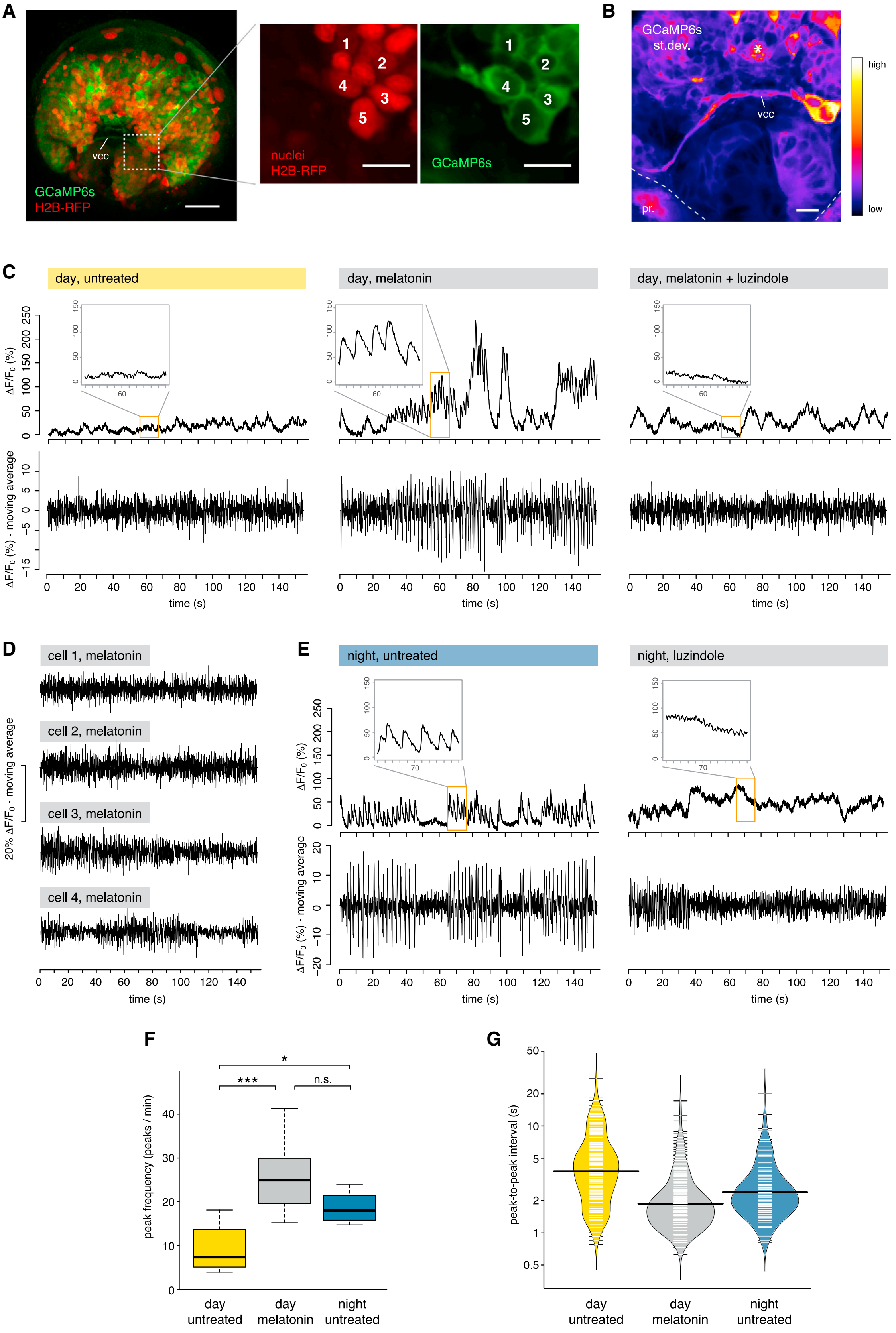

(legend on next page)

clock-controlled, melatonin-releasing system. The expression of multiple opsins in nonvisual photoreceptors of the most anterior (or apical) part of the larval nervous system has been documented in other protostomes and in deuterostomes (Ooka et al., 2010; Passamaneck et al., 2011). A shared feature of this so-defined larval "apical nervous system" (specified by conserved developmental patterning mechanisms; Marlow et al., 2014) is the presence of multiple sensory cells that project into a neurosecretory neuropil and release hormones and neuromodulators (Tosches and Arendt, 2013).

The expression of different opsins in subsets of *hiomt*+ cells in *Platynereis* hints at a sophisticated responsiveness of the apical nervous system to light. Opsins responding to different wavelengths would allow dissecting the spectral composition of light at different depths or during dusk and dawn and may also contribute to moonlight detection for the regulation of circalunar rhythms, which in adult worms has been attributed to the brain region harboring the ciliary photoreceptors (Zantke et al., 2013). The upregulation of *peropsin*, *neuropsin*, and *CNG* channels during the dark phase might then prepare the larvae to sense moonlight. The expanding array of functional tools available for *Platynereis* (Zantke et al., 2014) will allow testing these hypotheses and unraveling the contributions of individual opsins and neuropeptides to the annelid ambient light-dependent behaviors.

A Cholinergic Ciliomotor System Mediating a Circadian Behavioral State Switch

On the effector side, we identify a pair of cholinergic ciliomotor neurons as regulators of ciliary arrests. These neurons belong to a larger cholinergic system that innervates larval ciliary bands, as revealed by acetylcholinesterase staining; within this system, they are the only neurons expressing the melatonin receptor and responding to melatonin treatments. Cholinergic innervation of ciliary bands has been observed in most protostome and deuterostome larvae, and a general requirement of cholinergic transmission for ciliary arrests has been also documented (Lacalli and Gilmour, 1990; Lacalli et al., 1990), indicating that the cholinergic ciliomotor system described here is of broader relevance for understanding zooplankton neurobiology.

In *Platynereis* larvae, the melatonin-sensitive cholinergic system regulates distinct, long-lasting diurnal and nocturnal behavioral states. At the level of the ciliomotor neurons, this regulation involves the switch from sparse firing to rhythmic bursting. This finding is especially interesting as it may shed light on the evolutionary origin of circadian behavioral states in animals. Circadian behavioral states, such as sleep-wake cycles, are widespread in the animal kingdom, indicating that the day-night regulation of activity rhythms is crucial for homeostasis and survival (Allada and Siegel, 2008). A defining and conserved feature of sleep, which makes it distinct from rest, is the disconnection of the brain from the sensory environment and hence the increase of arousal threshold. The function of this sensory disconnection is still mysterious. *Platynereis* larvae can respond to a variety of sensory cues, which would ultimately affect ciliary arrests through the same ciliomotor system. For example, ciliomotor neurons are innervated by interneurons of the larval visual system, indicating that they respond to visual stimuli, together with other motoneurons (Randel et al., 2014). But could any sensory cue be relayed in presence of rhythmic bursting? If not, the nocturnal rhythmic burst firing might be a mechanism that filters incoming sensory information and reduces responsiveness to sensory stimuli. In a similar way, in mammals, the switch of thalamic relay neurons to rhythmic burst firing is responsible for filtering sensory information during sleep (McCormick and Feeser, 1990) and can be induced directly by melatonin (Ochoa-Sanchez et al., 2011). It will be interesting to determine whether these functional analogies correspond to conserved molecular mechanisms, for instance, to conserved targets downstream of melatonin signaling. Promising candidates are Shaker voltage-gated potassium channels, key determinants of neuronal excitability, which are regulated by melatonin (Yang et al., 2011) and are necessary for sleep in flies and mammals (Cirelli et al., 2005; Douglas et al., 2007).

Early Evolution of Melatonin Signaling in the Marine Environment

In marine zooplankton, the most prominent behavior associated with day-night cycles is DVM (see Introduction). We

Figure 5. Melatonin-Dependent Rhythmic Activity in Ventral Cholinergic Neurons

(A) Left: expression of GCaMP6s (green) and H2B-RFP (red) in the brain of larvae injected with GCaMP6s and H2B-RFP mRNAs at two-cell stage (Z-projection of the episphere, apical view). Scale bar, 30 μm. Middle and right: magnification of the most ventral cells of the CD lineage labeled by GCaMP6s and H2B-RFP. These cells correspond to ventral brain cholinergic neurons (compare Figure S4). Scale bar, 10 μm.

(B) SD of GCaMP6s fluorescence (apicoventral view) after melatonin treatment in a 2-min-long recording, showing that the most ventral neuron of the CD domain (compare cell number 5 in A) responds to melatonin with dynamic activity (high SD) and projects to the prototroch (pr, dashed line) through the ventral cerebral commissure (vcc). Scale bar, 10 μm.

(C) GCaMP6s fluorescence of a representative prototroch-projecting cholinergic neuron (cell number 5 in A) during daytime, in control conditions (left), after treatment with 100 μM melatonin (middle), and after subsequent addition of 100 μM of the melatonin receptor antagonist luzindole (right). Upper row: GCaMP6s signal normalized with a global F_0; lower row: GCaMP6s signal normalized with a local F_0 (moving average over a window of 0.8 s), to highlight changes in fluorescence over a small timescale (see the Experimental Procedures for details on the analysis).

(D) GCaMP6s fluorescence of the cells 1–4 (A) after treatment with melatonin. Data normalized with the moving average approach.

(E) GCaMP6s fluorescence of a representative prototroch-projecting cholinergic neuron at night, in control conditions (left), and after treatment with 100 μM luzindole (right).

(F) Frequency of GCaMP6s peaks (number of peaks/min) in untreated larvae during daytime (yellow), after melatonin treatment during daytime (gray), and in untreated larvae during the night (blue). n = 10, 8, and 4 larvae, respectively. One-way ANOVA with post hoc Holm adjustment, *$p < 0.05$, ***$p < 0.001$.

(G) Bean plot showing the distribution (on the logarithmic scale) of the peak-to-peak intervals (s) in untreated larvae during daytime (yellow), after melatonin treatment during daytime (gray), and in untreated larvae at night (blue). Thin white lines indicate single data points; black horizontal lines indicate means. n = 10, 8, and 4 larvae, respectively.

See also Figures S4 and S5 and Movie S1.

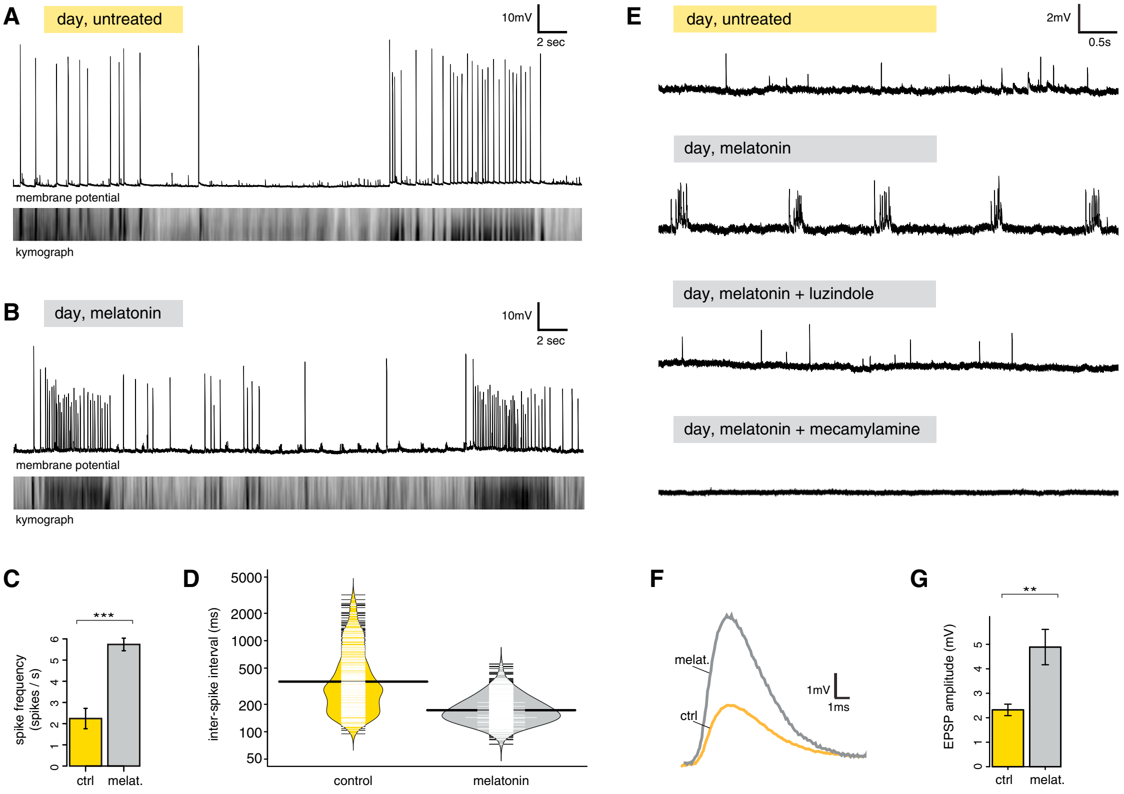

Figure 6. Melatonin Induces Rhythmic Bursting of Cholinergic Prototroch Presynaptic Neurons and an Enhanced Release of ACh

(A) Intracellular recording from a prototroch cell during daytime (top), and kymograph of ciliary beating imaged simultaneously (bottom; black bars: ciliary arrests), showing the correlation of prototroch spikes with ciliary arrests (100 μM).

(B) Same as (A), in a melatonin-treated larva (100 μM).

(C) Average prototroch spike frequency in controls (NSW) and in melatonin-treated larvae. $p < 0.001$, n = 7 larvae each. Error bars, SEM.

(D) Bean plot showing the distribution (on the logarithmic scale) of interspike intervals (ms) in controls (NSW, yellow) and in melatonin-treated larvae (100 μM, gray). Thin white lines indicate single data points; black horizontal lines indicate means. n = 7 larvae each.

(E) EPSPs recorded from prototroch cells. Traces (from top to bottom) represent EPSPs in untreated larvae and EPSPs after treatment with melatonin (100 μM), melatonin plus subsequent addition of luzindole (100 μM), and melatonin plus subsequent addition of mecamylamine (100 μM).

(F) Average EPSP responses measured from prototroch cells in absence (yellow) and the presence of melatonin (gray). n = 7 larvae.

(G) Average EPSPs amplitude (mV) in controls (yellow) and melatonin-treated larvae (gray). n = 7 larvae, $p < 0.01$, paired t test. Error bars, SEM.

See also Figure S6.

propose that the day-night changes in ciliary closure time evoked by melatonin signaling in *Platynereis* correspond to different phases of DVM in the field. In *Platynereis*, a long ciliary closure time correlates with a low vertical position (Conzelmann et al., 2011); therefore, the nocturnal behavior would lead to the slow descent of larvae in the water column. In line with this conclusion, field studies have shown that planktonic annelids reach their highest position in the water column at dusk and then sink throughout the whole night (Alldredge and King, 1980). Other zooplankton species show different temporal DVM regimes that often involve muscular rather than ciliary swimming, suggesting a more complex regulation of locomotor activities downstream of melatonin signaling as an adaptation to variable ecological constraints (Lampert, 1989).

Interestingly, it has been proposed that circadian rhythms and ambient light detection evolved in the context of DVM, an advantageous behavior facilitating escape from light-induced oxidative stress (Gehring and Rosbash, 2003; Pittendrigh, 1993). Light-sensitive cryptochromes, present in all basal metazoan phyla, including sponges (Rivera et al., 2012), might have represented the first link between ambient light detection and clock entrainment. We now extend this reasoning with the idea that melatonin signaling has been added to this system as an efficient paracrine signal, ensuring better coordination of the organismal response and whole-body physiology and becoming a perfect "chemical indicator of darkness" after its original radical scavenger role. Because melatonin receptors and opsins arose from the same duplication of an ancestral G-protein-coupled receptor (Feuda et al., 2012), we further hypothesize that melatonin signaling and opsin-based phototransduction evolved in the same DVM context. As photopigment, opsin outperformed cryptochrome by increased signaling speed, signal amplification, coupling with different transduction cascades and an evolvable spectral tuning that, after repeated duplications, allowed perception of colors at different depths and times

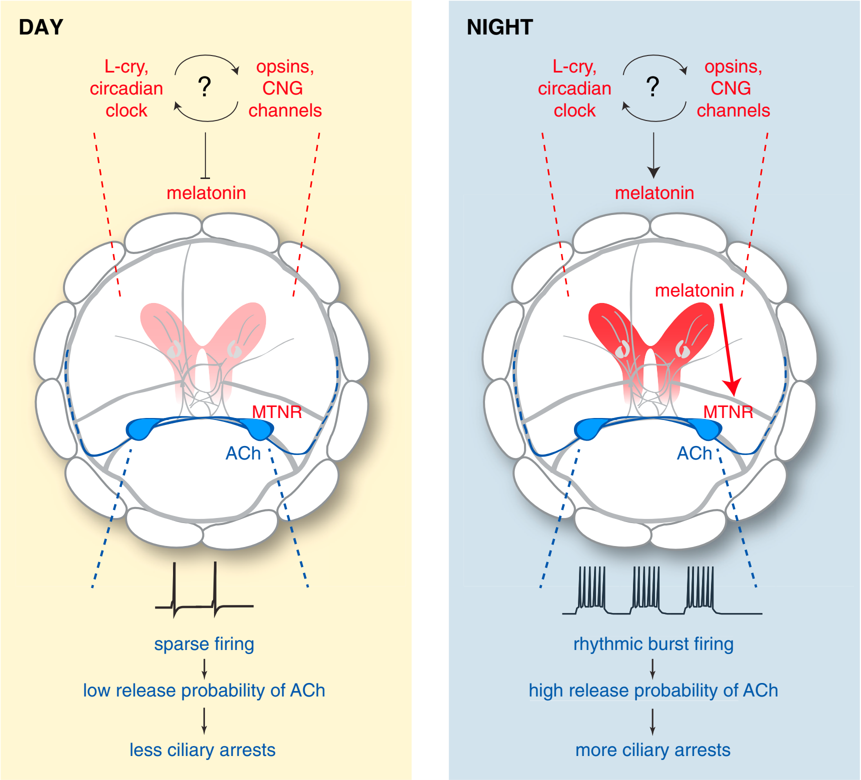

Figure 7. Day-Night Modulation of *Platynereis* Ciliary Swimming by Melatonin Signaling

In the extended ciliary photoreceptor region (red), the circadian clock and various opsins with a CNG-based phototransduction cascade regulate rhythmic release of melatonin. During the day (left), ventral cholinergic ciliomotor neurons (blue) fire sporadically and arrhythmically. Basal acetylcholine release at the ciliomotor-prototroch synapses ensures a low frequency and duration of ciliary arrests. At night (right), high melatonin levels directly affect the electrical activity of the cholinergic ciliomotor neurons, which express the *melatonin receptor* (*MTNR*). Therefore, the cholinergic neurons switch to a rhythmic bursting mode, which boosts the release of acetylcholine at the ciliomotor-prototroch synapses (i.e., increase of EPSPs amplitude). This increases the frequency of spiking in prototroch cells, causing longer and more frequent ciliary arrests.

of the day (Nilsson, 2013). This way the full system involving opsin-based ambient light detection, circadian clock, and melatonin signaling became a key regulator of circadian behavior and persisted in the dorsal brain of annelids and in the pineal and retinal photoreceptors of vertebrates.

EXPERIMENTAL PROCEDURES

Whole-Mount In Situ Hybridizations, Image Registration, and Acetylcholinesterase Staining

Platynereis genes were either obtained from expressed sequence tag libraries or cloned with rapid amplification of cDNA ends (RACE) PCRs and/or RT-PCR using gene specific primers (PCR primers are listed in the Extended Experimental Procedures). In situ hybridizations and acetylcholinesterase stainings (AChE) were performed and imaged following established protocols, as detailed in the Extended Experimental Procedures.

qRT-PCR Screen with Fluidigm Dynamic Arrays

Platynereis larvae were fertilized at ZT8 (midday) and raised in an 18°C incubator, under a 16L:8D cycle. Nine groups were sampled at different time points, as indicated in Figures 2 and S3. At night, animals were collected under dim red light. For the experiments with the inverted cycle conditions, immediately after fertilization, sibling larvae were raised under an inverted light cycle (12 hr shift).

cDNA was synthesized from 100 ng of total RNA; 1.25 μl of each cDNA was used as a template for a preamplification reaction (PreAmp) of target genes. Diluted PreAmps were loaded on a 48.48 Fluidigm BioMark Dynamic Array chip (Spurgeon et al., 2008). The chip was run following manufacturer's instructions (see the Extended Experimental Procedures for further details on the protocol and the list of primers used). Cq values were obtained with the Fluidigm Real-Time PCR Analysis software. Fluidigm data were analyzed with the Bioconductor HTqPCR package (Dvinge and Bertone, 2009). The genes *cdc5* and *rps9* were used to obtain the normalized dCq values, and for each time point the two *dCqs* from the two PreAmps were averaged. Expression relative to the first time point (45 hpf) was calculated as 2^{-ddCq}. The expression values of all the genes (except the housekeeping genes) were used for clustering analysis. Clustering was performed with the "average" method using the Euclidean distance. For clustering, expression values were scaled to the same range in order to compare similar trends regardless of the amplitude of expression changes. Comparable results were obtained from three biological replicas.

Behavioral Experiments

Behavioral experiments were typically performed between 51 and 55 hpf. The following drugs were used: L-*cis*-diltiazem (Enzo Life Sciences) in natural sea water (NSW), luzindole (Tocris), stock in DMSO, mecamylamine (Sigma), stock in 95% EtOH or NSW, and melatonin (Sigma) and acetylcholine (Sigma) solutions freshly prepared in NSW. Drugs were diluted to their final concentration in NSW; corresponding concentrations of vehicle were used as controls. Ciliary beating and arrests were imaged as described (Conzelmann et al., 2011), with a DMK 21BF04 camera (The Imaging Source) and a frame rate of 60 or 15 frames/s, respectively. A 750-nm-long pass filter was always interposed between the light source and larvae. Ciliary closure time was measured from 1-min-long movies. Behavioral data were analyzed in R. All the experiments were repeated at least twice with larvae from different embryonic batches.

Two-Photon Calcium Imaging with GCaMP6s

Platynereis CD blastomeres were injected with GCaMP6s (Chen et al., 2013) and H2B-RFP mRNAs (final concentration of 250 μg/μl each). Injected larvae at the 48–52 hpf stage were mounted in 3% low-melting agarose on glass-bottom culture dishes (MatTek) and maintained in NSW. Imaging was performed using a Zeiss LSM780 microscope, with a 32-Ch GaAsP detector and a two-photon light source (Chamaleon, Coherent) set at 910 nm. Two-photon imaging was chosen to avoid any interference of light with behavioral responses. Movies (248 × 250 pixels) were acquired under a 40× oil-immersion objective and with a temporal resolution between 8.26 and 16 Hz. Responses to drugs were imaged 5–20 min after application to the bath and without any change of imaging settings. GCaMP6s movies were analyzed using FIJI and R, as described in the Extended Experimental Procedures. The same region of interest (ROI) was used to quantify fluorescence before and after drug application. Data are presented as $\Delta F/F_0$.

Cell

Electrophysiology

Sharp electrode recordings with simultaneous high-speed imaging were performed on 40–60 hpf *Platynereis* larvae. Holding pipettes were made from borosilicate glass (Science Products) with an outer diameter (od) of 1 mm and were fire polished to minimize damage to the larvae. Recording electrodes were made from pipettes with an od of 1.5 mm, filled with 3 M KCl, and showed resistances between 15–25 mΩ. To facilitate electrode placement, larvae were digested with 46.7 μg/ml of Proteinase K for 10–15 min.

Electrophysiological recordings were performed on a multiclamp 700A amplifier. Signals were acquired at 20 kHz and analyzed using Clampfit 10.3 (Molecular Devices). Input resistances of prototroch cells were monitored by delivering small hyperpolarizing currents via the recording electrode, and only prototroch cells that displayed resting potentials between −65 to −80 mV and input resistances between 10–25 mΩ were used for analysis. Simultaneous high-speed (20 Hz) imaging of ciliary beating was performed on an Andor Neo S-CMOs camera and analyzed using FIJI.

ACCESSION NUMBERS

The new sequences have been deposited in GenBank (accession numbers: KM199642 to KM199652 and KM393192 to KM393194).

SUPPLEMENTAL INFORMATION

Supplemental Information includes Extended Experimental Procedures, six figures, and one movie and can be found with this article online at http://dx.doi.org/10.1016/j.cell.2014.07.042.

ACKNOWLEDGMENTS

The work was supported by the European Molecular Biology Laboratory (to M.A.T., D.B., P.V., and D.A.), by a European Research Council grant (EA-AdG-2011-294810_BrainEvoDevo) (to D.A., M.A.T., and P.V.), and by Deutsche Forschungsgemeinschaft grants (AR 387/2-1 to D.A; SFB488-A14 to D.A. and D.B.). We thank the EMBL Advanced Light Microscopy Facility and Genomic Core Facility, with special thanks to P. Collier and V. Benes for the Fluidigm experiments, H. Bading for generous access to the resources of the Department of Neurobiology, IZN, Heidelberg, C.P. Bengtson for assistance during electrophysiology experiments, G. Jekely for the initial analysis of *MTNR* and *CNGAα* expression and critical feedback, K. Tessmar-Raible for insightful discussions, H. Martinez-Vergara for producing the GCaMP6s mRNA, R. Tomer for averaging gene expression patterns for PrImR, H. Roebert for the initial efforts to clone the *hiomt* gene, and the Arendt lab for constructive feedback.

Received: March 13, 2014
Revised: June 7, 2014
Accepted: July 25, 2014
Published: September 25, 2014

REFERENCES

Allada, R., and Siegel, J.M. (2008). Unearthing the phylogenetic roots of sleep. Curr. Biol. *18*, R670–R679.

Alldredge, A.L., and King, J.M. (1980). Effects of moonlight on the vertical migration patterns of demersal zooplankton. J. Exp. Mar. Biol. Ecol. *44*, 133–156.

Anctil, M., Pani, A.K., and Ali, M.A. (1991). Modulation of rhythmic contractions by melatonin via cyclic GMP in the coelenterate *Renilla koellikeri*. J. Comp. Physiol. B *161*, 569–575.

Arendt, D., Tessmar-Raible, K., Snyman, H., Dorresteijn, A.W., and Wittbrodt, J. (2004). Ciliary photoreceptors with a vertebrate-type opsin in an invertebrate brain. Science *306*, 869–871.

Balzer, I., and Hardeland, R. (1991). Photoperiodism and effects of indoleamines in a unicellular alga, *Gonyaulax polyedra*. Science *253*, 795–797.

Bentkowski, P., Markowska, M., and Pijanowska, J. (2010). Role of melatonin in the control of depth distribution of *Daphnia magna*. Hydrobiologia *643*, 43–50.

Calkins, J., and Thordardottir, T. (1980). The ecological significance of solar UV radiation on aquatic organisms. Nature *283*, 563–566.

Chen, T.-W., Wardill, T.J., Sun, Y., Pulver, S.R., Renninger, S.L., Baohan, A., Schreiter, E.R., Kerr, R.A., Orger, M.B., Jayaraman, V., et al. (2013). Ultrasensitive fluorescent proteins for imaging neuronal activity. Nature *499*, 295–300.

Cirelli, C., Bushey, D., Hill, S., Huber, R., Kreber, R., Ganetzky, B., and Tononi, G. (2005). Reduced sleep in Drosophila Shaker mutants. Nature *434*, 1087–1092.

Conzelmann, M., Offenburger, S.-L., Asadulina, A., Keller, T., Münch, T.A., and Jékely, G. (2011). Neuropeptides regulate swimming depth of *Platynereis* larvae. Proc. Natl. Acad. Sci. USA *108*, E1174–E1183.

Dollins, A.B., Zhdanova, I.V., Wurtman, R.J., Lynch, H.J., and Deng, M.H. (1994). Effect of inducing nocturnal serum melatonin concentrations in daytime on sleep, mood, body temperature, and performance. Proc. Natl. Acad. Sci. USA *91*, 1824–1828.

Douglas, C.L., Vyazovskiy, V., Southard, T., Chiu, S.-Y., Messing, A., Tononi, G., and Cirelli, C. (2007). Sleep in Kcna2 knockout mice. BMC Biol. *5*, 42.

Dvinge, H., and Bertone, P. (2009). HTqPCR: high-throughput analysis and visualization of quantitative real-time PCR data in R. Bioinformatics *25*, 3325–3326.

Falcón, J., Besseau, L., Manganou, E., Sauzet, S., Fuentès, M., and Boeuf, G. (2010). The pineal organ of fish. In Biological Clock in Fish, E. Kulczykowska, W. Popek, and B.G. Kapoor, eds. (Boca Raton, FL: CRC Press).

Feuda, R., Hamilton, S.C., McInerney, J.O., and Pisani, D. (2012). Metazoan opsin evolution reveals a simple route to animal vision. Proc. Natl. Acad. Sci. USA *109*, 18868–18872.

Forward, R.B. (1988). Diel vertical migration: zooplankton photobiology and behaviour. Oceanogr. Mar. Biol. Ann. Rev. *26*, 361–393.

Gehring, W., and Rosbash, M. (2003). The coevolution of blue-light photoreception and circadian rhythms. J. Mol. Evol. *57* (*Suppl 1*), S286–S289.

Hardeland, R., and Poeggeler, B. (2003). Non-vertebrate melatonin. J. Pineal Res. *34*, 233–241.

Hardeland, R., Balzer, I., Poeggeler, B., Fuhrberg, B., Uría, H., Behrmann, G., Wolf, R., Meyer, T.J., and Reiter, R.J. (1995). On the primary functions of melatonin in evolution: mediation of photoperiodic signals in a unicell, photooxidation, and scavenging of free radicals. J. Pineal Res. *18*, 104–111.

Jékely, G., Colombelli, J., Hausen, H., Guy, K., Stelzer, E., Nédélec, F., and Arendt, D. (2008). Mechanism of phototaxis in marine zooplankton. Nature *456*, 395–399.

Jiang, Z.G., Nelson, C.S., and Allen, C.N. (1995). Melatonin activates an outward current and inhibits Ih in rat suprachiasmatic nucleus neurons. Brain Res. *687*, 125–132.

Lacalli, T.C., and Gilmour, T.H.J. (1990). Ciliary reversal and locomotory control in the pluteus larva of *Lytechinus pictus*. Philos. Trans. R. Soc. London. Ser. B *330*, 391–396.

Lacalli, T.C., Gilmour, T.H.J., and West, A.E. (1990). Ciliary band innervation in the bipinnaria larva of *Piaster ochraceus*. Philos. Trans. R. Soc. London. Ser. B *330*, 371–390.

Lamb, T.D. (2013). Evolution of phototransduction, vertebrate photoreceptors and retina. Prog. Retin. Eye Res. *36*, 52–119.

Lampert, W. (1989). The adaptive significance of diel vertical migration of zooplankton. Funct. Ecol. *3*, 21–27.

Marlow, H., Tosches, M.A., Tomer, R., Steinmetz, P.R., Lauri, A., Larsson, T., and Arendt, D. (2014). Larval body patterning and apical organs are conserved in animal evolution. BMC Biol. *12*, 7.

McCormick, D.A., and Feeser, H.R. (1990). Functional implications of burst firing and single spike activity in lateral geniculate relay neurons. Neuroscience *39*, 103–113.

Nilsson, D.-E. (2013). Eye evolution and its functional basis. Vis. Neurosci. *30*, 5–20.

Nowak, J.Z., Szymańska, B., Zawilska, J.B., and Białek, B. (1993). Hydroxyindole-O-methyltransferase activity in ocular and brain structures of rabbit and hen. J. Pineal Res. *15*, 35–42.

Ochoa-Sanchez, R., Comai, S., Lacoste, B., Bambico, F.R., Dominguez-Lopez, S., Spadoni, G., Rivara, S., Bedini, A., Angeloni, D., Fraschini, F., et al. (2011). Promotion of non-rapid eye movement sleep and activation of reticular thalamic neurons by a novel MT2 melatonin receptor ligand. J. Neurosci. *31*, 18439–18452.

Ooka, S., Katow, T., Yaguchi, S., Yaguchi, J., and Katow, H. (2010). Spatiotemporal expression pattern of an encephalopsin orthologue of the sea urchin *Hemicentrotus pulcherrimus* during early development, and its potential role in larval vertical migration. Dev. Growth Differ. *52*, 195–207.

Passamaneck, Y.J., Furchheim, N., Hejnol, A., Martindale, M.Q., and Lüter, C. (2011). Ciliary photoreceptors in the cerebral eyes of a protostome larva. Evodevo *2*, 6.

Pittendrigh, C.S. (1993). Temporal organization: reflections of a Darwinian clock-watcher. Annu. Rev. Physiol. *55*, 16–54.

Randel, N., Bezares-Calderón, L.A., Gühmann, M., Shahidi, R., and Jékely, G. (2013). Expression dynamics and protein localization of rhabdomeric opsins in *Platynereis* larvae. Integr. Comp. Biol. *53*, 7–16.

Randel, N., Asadulina, A., Bezares-Calderón, L.A., Verasztó, C., Williams, E.A., Conzelmann, M., Shahidi, R., and Jékely, G. (2014). Neuronal connectome of a sensory-motor circuit for visual navigation. eLife, e02730.

Reiter, R.J. (1993). The melatonin rhythm: both a clock and a calendar. Experientia *49*, 654–664.

Rhode, S.C., Pawlowski, M., and Tollrian, R. (2001). The impact of ultraviolet radiation on the vertical distribution of zooplankton of the genus *Daphnia*. Nature *412*, 69–72.

Rivera, A.S., Ozturk, N., Fahey, B., Plachetzki, D.C., Degnan, B.M., Sancar, A., and Oakley, T.H. (2012). Blue-light-receptive cryptochrome is expressed in a sponge eye lacking neurons and opsin. J. Exp. Biol. *215*, 1278–1286.

Roopin, M., and Levy, O. (2012). Melatonin distribution reveals clues to its biological significance in basal metazoans. PLoS ONE *7*, e52266.

Rudjakov, J.A. (1970). The possible causes of diel vertical migrations of planktonic animals. Mar. Biol. *6*, 98–105.

Spurgeon, S.L., Jones, R.C., and Ramakrishnan, R. (2008). High throughput gene expression measurement with real time PCR in a microfluidic dynamic array. PLoS One *3*, e1662.

Tan, D.X., Manchester, L.C., Terron, M.P., Flores, L.J., and Reiter, R.J. (2007). One molecule, many derivatives: a never-ending interaction of melatonin with reactive oxygen and nitrogen species? J. Pineal Res. *42*, 28–42.

Tanaka, D., Furusawa, K., Kameyama, K., Okamoto, H., and Doi, M. (2007). Melatonin signaling regulates locomotion behavior and homeostatic states through distinct receptor pathways in *Caenorhabditis elegans*. Neuropharmacology *53*, 157–168.

Tilden, A.R., Brauch, R., Ball, R., Janze, A.M., Ghaffari, A.H., Sweeney, C.T., Yurek, J.C., and Cooper, R.L. (2003). Modulatory effects of melatonin on behavior, hemolymph metabolites, and neurotransmitter release in crayfish. Brain Res. *992*, 252–262.

Tomer, R., Denes, A.S., Tessmar-Raible, K., and Arendt, D. (2010). Profiling by image registration reveals common origin of annelid mushroom bodies and vertebrate pallium. Cell *142*, 800–809.

Tosches, M.A., and Arendt, D. (2013). The bilaterian forebrain: an evolutionary chimaera. Curr. Opin. Neurobiol. *23*, 1080–1089.

Yang, X.-F., Miao, Y., Ping, Y., Wu, H.-J., Yang, X.-L., and Wang, Z. (2011). Melatonin inhibits tetraethylammonium-sensitive potassium channels of rod ON type bipolar cells via MT2 receptors in rat retina. Neuroscience *173*, 19–29.

Zantke, J., Ishikawa-Fujiwara, T., Arboleda, E., Lohs, C., Schipany, K., Hallay, N., Straw, A.D., Todo, T., and Tessmar-Raible, K. (2013). Circadian and circalunar clock interactions in a marine annelid. Cell Reports *5*, 99–113.

Zantke, J., Bannister, S., Rajan, V.B.V., Raible, F., and Tessmar-Raible, K. (2014). Genetic and genomic tools for the marine annelid *Platynereis dumerilii*. Genetics *197*, 19–31.

Zhdanova, I.V., Wang, S.Y., Leclair, O.U., and Danilova, N.P. (2001). Melatonin promotes sleep-like state in zebrafish. Brain Res. *903*, 263–268.

Supplemental Information

Cell

EXTENDED EXPERIMENTAL PROCEDURES

Animals

Platynereis dumerilii larvae were obtained from an established culture at EMBL. Larvae were raised in natural seawater (NSW), at 18°C under a 16 hr light - 8 hr dark cycle.

Sources of *Platynereis* Genes and Phylogenetic Analysis

Genes

New *Platynereis* genes were cloned either directly, with RT-PCR and gene specific primers, or first with RACE PCRs using a mixed stages cDNA library (from 24hpf, 39hpf, 48hpf, 56hpf, 72hpf and 5 dpf larvae, Invitrogen GeneRacer Kit). PCR primers were designed on the basis of available transcriptome and EST data, and for amplification we used either the HotStart Taq Polymerase (QIAGEN) or the Phusion polymerase (New England BioLabs). PCR fragments were cloned in the pCRII-Topo vector (Invitrogen) and verified by sequencing.

The following RACE primers were used to isolate the *hiomt* cDNA sequence: TTGGAGCATAAATGATTGGGCTGATGG, AAGGTGGCGATATTCCTTCCCTGAACG, ATGCCTCCACCAGGGTTGATTTTCTCA, GCTCAGCAGGTAGAGGTCGGCTTCA, TGAT GAAGAAGTCCCCGGGCAAA, TGCATGAGAGATACAGGGCCTTCGACA, AGTGCTGTCGATTTAGGAGCTGGCACA.

The following PCR primers were used to amplify cDNAs and produce probes for in situ hybridization:

Gene Name	Forward Primer	Reverse Primer
aanat	TGGTGTAGGTATAGCACAAGTCACATC	TTTTTATGTTGAAATTGAGTTTGGATTT
CNGAβ	ACTTGGATTGCAGGGTACTTGGAGCAA	ACATTATGACCATGGCGTTCCAACTGA
CNGB	CAGTCTGCAGATGCAGCGAAGACAAAT	TGTGAGCTTCAGGGTAGTCACTCATGG
gch	GACTCCAGCGACGTCAGTCCTATCCAT	GTTTGATTGGTGGACTGCACATTTTCG
Giα	ACAAATCTTTAAGACAGGATGGAG	GGTTCGTAACAACACTGTACC
hiomt	CAGGGGAAGGACGGAAAGCACAATTAC	CACCAAAACTTTGTGCACATATCACCTCA
mao	AGGAGCAGGCATAAGTGGTCTCTCAGC	CACAGTCAGAAAGCACACACTGGCAGA
neuropsin	TTCCCGGGATCCTGTTACCCAGTTAGG	GAGGGCTCTGACCGACTCTTGTTGTGA
peropsin	TGGTGATGTGCCACTGCTAGTCAGCAT	GCAAACTGCATAAACCAGAGGGACACCTG
vmat	TACTCGGACGACAAACAAAGGAGCAGA	GCAGCCATCTTGAAACGCACACATAAG
vrille	GGACACCACCAAGTGTGCCACTCC	ACTGTTTTCGCTTGTGGCGGTGTTACT

In order to design qPCR primers and/or produce probes for in situ hybridization, the sequences for the following genes were obtained from previous publications: *ddc* (Raible et al., 2005), *FMRF*, *phc2, TPH* and *vtn* (Tessmar-Raible et al., 2007), *ChAT* (Denes et al., 2007), *FVRI* (Jékely et al., 2008), *DLamide, FLamide, FVamide, YFamide, RYamide* and *WLDamide* (Conzelmann et al., 2011), *r-opsin4* (Randel et al., 2013), *tr-cry* and *clock* (Zantke et al., 2013) and *NPY-4* (Conzelmann et al., 2013).

Phylogenetic Analyses

Sequence data were retrieved from the JGI genome portal webserver, NCBI and ENSEMBL. Multiple sequence alignments of protein sequences were generated with MUSCLE (http://www.ebi.ac.uk/Tools/msa/muscle/) and with webPRANK (Löytynoja and Goldman, 2008), inspected and corrected in Jalview (Waterhouse et al., 2009), and trimmed with Gblocks (Castresana, 2000). Phylogenetic trees with the maximum likelihood method were computed with PhyML 3.0 (Guindon et al., 2010), with 100x bootstrap. Phylogenetic trees were plotted with FigTree.

Imaging of Gene Expression Patterns

In Situ Hybridizations

Whole mount in situ hybridizations and tubulin counterstaining were performed as described previously (Arendt et al., 2001), with few modifications. For *hiomt* in situs, probe binding specificity was improved by carrying hybridization and post-hybridization washes at 62°C and by using a lower SSC concentration for the last post-hybridization washes (0.1x SSC instead of 0.2x SSC). In situ hybridizations were normally counterstained with an anti-acetylated tubulin antibody (Sigma, T7451), which labels the axonal scaffold and the ciliary bands. For early developmental stages (<48hpf) or for weakly expressed genes, larvae were acetylated after the proteinase K digestion with the following protocol: 5 min in 1% triethanolamine, 5 min in 1% triethanolamine 3 μl/ml acetic anhydride, 5 min in 1% triethanolamine 6 μl/ml acetic anhydride. Acetylation was found to improve staining speed and signal-to-noise ratio. Acetylated larvae were counterstained with the anti-tyrosinated tubulin antibody (Sigma T9028).

Imaging

We used a Zeiss Axio Imager.M1 microscope for light microscopy and a Leica TCS SPE and a Leica TSC SP8 for confocal microscopy. Confocal stacks of expression patterns were acquired with reflection microscopy (Jékely and Arendt, 2007), using a 40x oil immersion objective. Images were processed in Fiji (Schindelin et al., 2012). Contrast and brightness were adjusted equally throughout the images.

Image Registration

Profiling by Image Registration (PrImR) was performed as described in Tomer et al. (2010) and Asadulina et al. (2012). In silico coexpression analysis from PrImR average expression patterns was performed using built-in functions in Fiji and Imaris.

Acetylcholinesterase Staining and Imaging

Acetylcholinesterase (AChE) staining was performed according to Denes et al. (2007) and Karnovsky and Roots (1964), with some modifications. Before fixation, larvae were digested for 1 min with 46.7 μg/ml of proteinase K in NSW. Larvae were then fixed in ice-cold EtOH for 2 min or in 4% PFA for 10 min, and stained for 3-4 hr at room temperature in 65mM phosphate buffer pH 6, 0.5mg/ml ACh iodide, 5mM sodium citrate, 3mM copper sulfate and 0.5mM potassium ferricyanide. Staining was stopped with 50% EtOH, then larvae were dehydrated with an EtOH series and transferred in 87% glycerol for imaging. Reflection microscopy was used for confocal imaging of the AChE signal.

qRT-PCR Screen with Fluidigm Dynamic Arrays

Sample Preparation

Eight batches of *Platynereis* larvae were fertilized at the same time at ZT8 (midday). They were subsequently pooled, separated again into nine groups and raised in a 18°C incubator, under a 16 hr light - 8 hr dark cycle. The nine groups were sampled at different time points between 45hpf and 75hpf, and frozen in liquid nitrogen. The time points were selected as follows (see also Figure 2A): 1) 45hpf ZT5; 2) 48hpf ZT8; 3) 54hpf ZT14; 4) 57hpf ZT17; 5) 60hpf ZT20; 6) 63.5hpf ZT23.5; 7) 67.5hpf ZT3.5; 8) 72.5hpf ZT8.5; 9) 75hpf ZT11. For the nighttime samples, animals were collected under dim red light. For the experiments with the inverted cycle conditions, immediately after fertilization sibling larvae were transferred into a different 18°C incubator with inverted light cycle conditions (12 hr shift), and then sampled at the same developmental stages. Total RNA was extracted from frozen samples using Trizol (Peqlab) and phenol-chloroform, and RNA quality was assessed with Bioanalyzer (Agilent Technologies).

Reverse Transcription, Preamplification of cDNA, and qRT-PCR

For each sample, 100ng of total RNA were reversed transcribed with SuperscriptIII First-Strand Synthesis SuperMix for qRT-PCR (Invitrogen), in a 10 μl reaction with oligo-d(T) and random hexameres. 1.25 μl of each cDNA (corresponding to 12.5ng of initial RNA) were used as template for a preamplification reaction (PreAmp) with the ABI PreAmp Mastermix Kit (PN4391128). For each sample, PreAmps were run in duplicate.

Each PreAmp consisted in the specific target amplification (STA) of all the 48 target genes. The gene specific primers were pooled to a final concentration of 50nM each, and preamplification was carried out for 14 cycles. After PreAmp, STA primers were removed with Exonuclease I (NEB), and the cDNAs were diluted 10 times. Conventional qPCR was used to confirm the linearity of the PreAmp reactions.

To quantify gene expression levels, we used a high-throughput microfluidics qPCR system from Fluidigm (Spurgeon et al., 2008). 48.48 Fluidigm BioMark Dynamic Array chips were used to measure the expression of 48 target genes, following the manufacturer instructions. The inlets were loaded with 2.25 μl of the diluted PreAmp cDNAs or with gene specific primers, to an inlet concentration of 5 μM (corresponding to a 500nM concentration in the final reaction). Serial dilutions of the templates were included to control the linearity of the amplification. The Fluidigm Real Time PCR Analysis software was used to set an optimal global threshold for all the target genes and obtain Cq values. These results were confirmed with independent qPCR experiments on a subset of target genes.

Data Analysis

The Fluidigm data were analyzed with the Bioconductor HTqPCR package (Dvinge and Bertone, 2009). The genes *cdc5* and *rps9*, previously shown as reliable normalization genes (Dray et al., 2010), were used to obtain the normalized *dCq* values, and for each time point the two *dCqs* from the two PreAmps were averaged. Expression relative to the first time point (45hpf) was calculated as 2^{-ddCq}. The expression values for all the genes (except the housekeeping genes) were used for clustering analysis. Clustering was performed with the "average" method using the Euclidean distance. For clustering, expression values were scaled in order to compare similar trends regardless of the amplitude of expression changes. Figures 2 and S3 show one representative biological replica. We obtained comparable results for other two biological replicates assayed independently.

qPCR Primers

qPCR primers were designed to target, whenever possible, sequences without SNPs, and to span exon-exon junctions (as assessed from internal transcriptomic and genomic resources). The efficiency of all the primer pairs was determined experimentally using as template a dilution series of cDNA; only primers with efficiency between 90% and 110% were used. The selected primers have an average Tm of 60°C and an average amplicon size of 91.4bp. All the primers used for qPCR are listed in the following table (gene name, forward primer, reverse primer):

Gene Name	Forward qPCR Primer	Reverse qPCR Primer
cdc5	CCTATTGACATGGACGAAGATG	TTCCCTGTGTGTTCGCAAG
rps9	CGCCAGAGAGTTGCTGACT	ACTCCAATACGGACCAGACG
aanat	CCAAACATCATGGCACTGAC	AGTGGCAGCTTCATCACTTG

(*Continued on next page*)

Continued		
Gene Name	Forward qPCR Primer	Reverse qPCR Primer
ddc	ATGTTGTTCCGACCGATGAC	GAAAGCACATGTCGGAGTTG
gch	AAAGCCCTCCTCTACTTCACC	TCTCGGACAATCACCATCTC
hiomt	ACATGTGGGACACCTTCATC	AGGTGGCGATATTCCTTCC
mao	ATCTGGGAGGTCGATAATGG	GCTGACTTGCATTGAACCTC
MTNR	ATCAATCCCTTCAGCATTACAG	CGACAGAGGCCCAAATTATC
sert	CAACGAGACCAACGTCAAAC	CCCAAGCCTCTTGCTTTATC
TPH	CCCTCAAGAAGCTGAATCTG	ATTTCGAGAGTTGGGGTCTC
vmat	GATGGTTATCAGCCGGATTC	GAATGGGATACAAGCCAAGC
bmal	CAGCATGCCATAAAAAGAAACG	CAGGATTTCAAATAACCAGCAAAA
clock	GAGGTTCCCGAATATTCAAATC	AGTCAGAGAGATCGGTTCGTAG
L-cry	AGAAGCCCTTCAACAAGTGG	GCGTTCAATCATGAGACGAG
tr-cry	ATGGGACAAGAATCCAGAGG	CATGACGAGCAAGATGATGG
vrille	ACTGGATGAAGAGACGCAAG	TTAGCTCACGACGAAGGTTG
period	TCCTGCAGCACATGAAGAAG	TGGCACCAAGGAGTTCTATG
c-ops1	TCTGCAAGTGGTATGGCTTC	AAACTTCCAAGCCTCAGGAC
neurops	ATTTCGCCGAGAGGTGATAG	TCGCCATGGTTGCATTAG
perops	CGAGGTATGCTTTGGACAAG	GAAGCAGCTCATGGTTATGG
r-ops1	ACCCTATGCCGTCGTTGGA	GGCGAGCAGAGGGTGGAT
r-ops3	TCATTCACTGGGTCATAGGG	TGGACGGAGTTCGTAATGAG
r-ops4	AAAGCCATCCAGCACTCAC	CAATAGCCAGATGGGTTGAG
bsx	CCCAGAAAGAGTGGAATTGG	TGCGGTGAATGTGACTGTTC
not	AAAGTCAGGAGGGATGGTTC	CCTAACCCTCTTTGCTTTGC
DLamide	GCATTGCCTTACAGAAGCAG	TGAGAAGACACTGACGACCAG
FLamide	CACACTTAAAGGAGGCAAGC	GAGCCTACATTGGCACTTTG
FMRF	ATTCGGGAAACGGGAAAG	CTTCCCAAACCTCATGAACC
FVamide	CTCACTAGGTGGGCAAAATG	TCATCTTCGTCATCCTCTCG
FVRI	CAAAGACCGACTTCACCAAG	CATACTGTCACAACGGACTGG
YFamide	CAATGGTGAACTGCAAGACC	GGATATCTCTTCTCGCTGTCG
NPY-4	GCAAGAGGACAATAGCAGAGTG	GTGGATATGTTCCATGGCTTC
PDF	AGGATGGGATCAAGCAAGAC	AGACAACCTGCATGACGAAC
RYamide	CTCTCATTCTCGCTTTAGCC	CCTCGCTTGTCATCTTCATC
vtn	CAGTGTTTCGGACCCAACAT	TAACGTGTTAATGTAGCATCCTATTGA
WLDamide	AAACGCCGTCCTAACTCAAG	GTGGCAGTTTGTTTGCTGTG
CNGAβ	TTGCTGGTCACAGAAAGAGG	AATCAGTGGGAAGGATGGAC
CNGAα	TGCCGTCCATAGCAGTAAAC	CAAAGCGCACTACAAAGCTG
CNGB	AGTCCTGCCTAAGATCCTGAAG	AAGAGCTGCACTTTGCTGAG
phc2	CAACGGAGAACACAACTGGA	CATATCCGAAGAGGTGGTTGA
syt	AAAGAAAAGGTGCAGCCTGA	TCATAGATGGCGAACACCAG

Behavioral Experiments

Animals

For all the behavioral experiments, animals were raised in an 18°C incubator, under a 16 hr light - 8 hr dark cycle. All experiments were typically performed between 51 and 55hpf. For the midday conditions (ZT8), fertilizations were set up at ZT4, whereas for midnight conditions (ZT20) fertilizations were set up at ZT16. Therefore the night experiments were specifically performed in the second half of the night phase.

Pharmacology

The following drugs were used: L-*cis*-diltiazem (Enzo Life Sciences), 10mM stock in NSW; luzindole (Tocris), 100mM stock in DMSO; mecamylamine (Sigma), 10mM stock in 95% EtOH or freshly dissolved in NSW; melatonin (Sigma) and ACh (Sigma) solutions were freshly prepared in NSW. The drugs were diluted to the final concentration in NSW; corresponding concentrations of vehicle were used as controls.

Imaging
Ciliary beating and ciliary arrests were imaged as described (Conzelmann et al., 2011), with a Zeiss Axiophot microscope equipped with a DMK 21BF04 camera (The Imaging Source). Movies were acquired with a frame rate of 60 or 15 frames/s, respectively. A 750nm long pass filter was always interposed between the light source and larvae. Ciliary closure time was measured from 1 min long movies. For the nighttime measurements, larvae were quickly mounted under a dim red light and imaging was performed in complete darkness. For the ACh experiments (Figure 4), each larva was imaged after acute treatment with ACh and/or mecamylamine (within 1 min after drug application). For the melatonin and luzindole experiments, larvae were incubated with the drugs for at least 20 min before mounting and imaging.

Data Analysis
Behavioral data were analyzed in R (R Core Team, 2012). All the experiments were repeated at least twice with larvae from different embryonic batches. For the analysis of closure length distribution, the Log10 transformed data set was used in the histograms of Figure 3, and the data are normalized according to the number of larvae imaged in each condition.

Two-Photon Calcium Imaging with GCaMP6s
Microinjection of Zygotes
Platynereis zygotes were injected using a Zeiss Axiovert 40C inverted microscope, equipped with a joystick micromanipulator (Narishige) and a microinjector (FemtoJet, Eppendorf). Injection needles were pooled from glass capillaries (borosilicate thin wall with filament, 1.0 mm outer diameter, 0.78 mm inner diameter, Harvard apparatus) using a Sutter needle pooler. Embryos were accommodated on an agarose stage, customized for *Platynereis* injections.

GCaMP6s (Chen et al., 2013) was kindly provided by the GENIE Project, Janelia Farm Research Campus, Howard Hughes Medical Institute (Addgene plasmid 40753). The GCaMP6s coding sequence was subcloned in the pCS2+ vector, and mRNA was in vitro transcribed with the SP6 mMessage mMachine High Yield Capped RNA Transcription Kit (Ambion). GCaMP6s mRNA was injected in the CD blastomere to a final concentration of 250 μg/μl. H2B-RFP mRNA was co-injected at the same concentration in order to label the CD lineage.

Two-Photon Imaging
For calcium imaging, 48-52hpf larvae were mounted on glass bottom culture dishes (MatTek) in 3% low melting agarose, and maintained in natural seawater. Imaging was performed using a Zeiss LSM780 microscope, with a 32-Ch GaAsP detector and a two-photon light source (Chamaleon, Coherent) running at 910nm. Movies (248x250 pixels) were acquired under a 40x oil-immersion objective, for at least one minute and with a temporal resolution ranging from 8.26Hz to 16Hz. Drugs were applied directly to the seawater, and responses were imaged 20 min after application (or 5 min after application for the mecamylamine experiment in Figure S5). For the calcium imaging experiments during the night, larvae were mounted under dim red light and imaged in complete darkness.

Image Analysis
GCaMP6s movies were analyzed using FIJI and R. The cholinergic ciliomotor neuron was identified on the basis of its position within the CD domain and the presence of a contralateral projection. ROIs (region of interest) were manually selected around the cell body and/or the axon of the cholinergic ciliomotor neuron. In each experiment, the same ROI was used to compare GCaMP6s fluorescence before and after the drug treatments. For the calculation of normalized $\Delta F/F_0$ data with a global baseline, F_0 was set as the average fluorescence of the 11-frames interval centered on the minimum fluorescence value. For the calculation of normalized $\Delta F/F_0$ data with the moving average approach, a sliding window of 0.8 s was used to calculate F_0. For the analysis of peak frequency and peak-to-peak intervals, peaks were extracted from the imaging data with custom-written algorithms.

Electrophysiology
Sharp electrode recordings along with simultaneous high-speed imaging were performed on 40–60 hpf *Platynereis* larvae. Holding pipettes were made from borosilicate glass (Science Products GmbH, Hofheim) with an outer diameter (o.d.) of 1 mm and were fire polished to minimize damage to the larvae. Recording electrodes were made from pipettes with an o.d. of 1.5 mm, filled with 3M KCl, and showed resistances between 15 – 25 mΩ. To facilitate electrode placement, larvae were digested with 46.7 μg/ml of Proteinase K for 10-15 min.

Electrophysiological recordings were performed on a multiclamp 700A amplifier. Signals were acquired at 20 kHz and analyzed using Clampfit 10.3 (Molecular Devices, Union City, CA). Input resistances of prototroch cells were monitored by delivering small hyperpolarizing currents via the recording electrode and only prototroch cells which displayed resting potentials between −65 to −80 mV and input resistances between 10 – 25 mΩ were used for analysis. Simultaneous high-speed (20 Hz) imaging was performed on an Andor Neo S-CMOs camera and analyzed using FIJI.

SUPPLEMENTAL REFERENCES

Ackermann, C., Dorresteijn, A., and Fischer, A. (2005). Clonal domains in postlarval *Platynereis dumerilii* (Annelida: Polychaeta). J. Morphol. *266*, 258–280.

Anctil, M. (2009). Chemical transmission in the sea anemone *Nematostella vectensis*: A genomic perspective. Comp. Biochem. Physiol. Part D Genomics Proteomics *4*, 268–289.

Arendt, D., Technau, U., and Wittbrodt, J. (2001). Evolution of the bilaterian larval foregut. Nature *409*, 81–85.

Asadulina, A., Panzera, A., Verasztó, C., Liebig, C., and Jékely, G. (2012). Whole-body gene expression pattern registration in *Platynereis* larvae. Evodevo *3*, 27.

Castresana, J. (2000). Selection of conserved blocks from multiple alignments for their use in phylogenetic analysis. Mol. Biol. Evol. *17*, 540–552.

Conzelmann, M., Williams, E.A., Krug, K., Franz-Wachtel, M., Macek, B., and Jékely, G. (2013). The neuropeptide complement of the marine annelid *Platynereis dumerilii*. BMC Genomics *14*, 906.

Denes, A.S., Jékely, G., Steinmetz, P.R.H., Raible, F., Snyman, H., Prud'homme, B., Ferrier, D.E.K., Balavoine, G., and Arendt, D. (2007). Molecular architecture of annelid nerve cord supports common origin of nervous system centralization in Bilateria. Cell *129*, 277–288.

Dray, N., Tessmar-Raible, K., Le Gouar, M., Vibert, L., Christodoulou, F., Schipany, K., Guillou, A., Zantke, J., Snyman, H., Béhague, J., et al. (2010). Hedgehog signaling regulates segment formation in the annelid *Platynereis*. Science *329*, 339–342.

Guindon, S., Dufayard, J.-F., Lefort, V., Anisimova, M., Hordijk, W., and Gascuel, O. (2010). New algorithms and methods to estimate maximum-likelihood phylogenies: assessing the performance of PhyML 3.0. Syst. Biol. *59*, 307–321.

Jékely, G., and Arendt, D. (2007). Cellular resolution expression profiling using confocal detection of NBT/BCIP precipitate by reflection microscopy. Biotechniques *42*, 751–755.

Karnovsky, M.J., and Roots, L. (1964). A "direct-coloring" thiocholine method for cholinesterases. Mar. Histochem. Cytochem. *12*, 219–221.

Lacalli, T.C. (1984). Structure and organization of the nervous system in the trochophore larva of *Spirobranchus*. Philos. Trans. R. Soc. B Biol. Sci. *306*, 79–135.

Löytynoja, A., and Goldman, N. (2008). Phylogeny-aware gap placement prevents errors in sequence alignment and evolutionary analysis. Science *320*, 1632–1635.

R Core Team (2012). R: a language and environment for statistical computing. http://www.r-project.org/.

Raible, F., Tessmar-Raible, K., Osoegawa, K., Wincker, P., Jubin, C., Balavoine, G., Ferrier, D., Benes, V., de Jong, P., Weissenbach, J., et al. (2005). Vertebrate-type intron-rich genes in the marine annelid *Platynereis dumerilii*. Science *310*, 1325–1326.

Schindelin, J., Arganda-Carreras, I., Frise, E., Kaynig, V., Longair, M., Pietzsch, T., Preibisch, S., Rueden, C., Saalfeld, S., Schmid, B., et al. (2012). Fiji: an open-source platform for biological-image analysis. Nat. Methods *9*, 676–682.

Tessmar-Raible, K., Raible, F., Christodoulou, F., Guy, K., Rembold, M., Hausen, H., and Arendt, D. (2007). Conserved sensory-neurosecretory cell types in annelid and fish forebrain: insights into hypothalamus evolution. Cell *129*, 1389–1400.

Waterhouse, A.M., Procter, J.B., Martin, D.M., Clamp, M., and Barton, G.J. (2009). Jalview version 2—a multiple sequence alignment editor and analysis workbench. Bioinformatics *25*, 1189–1191.

Wilson, E.B. (1892). The cell lineage of *Nereis*. A contribution to the cytogeny of the annelid body. J. Morphol. *VI*, 361–478.

Cell

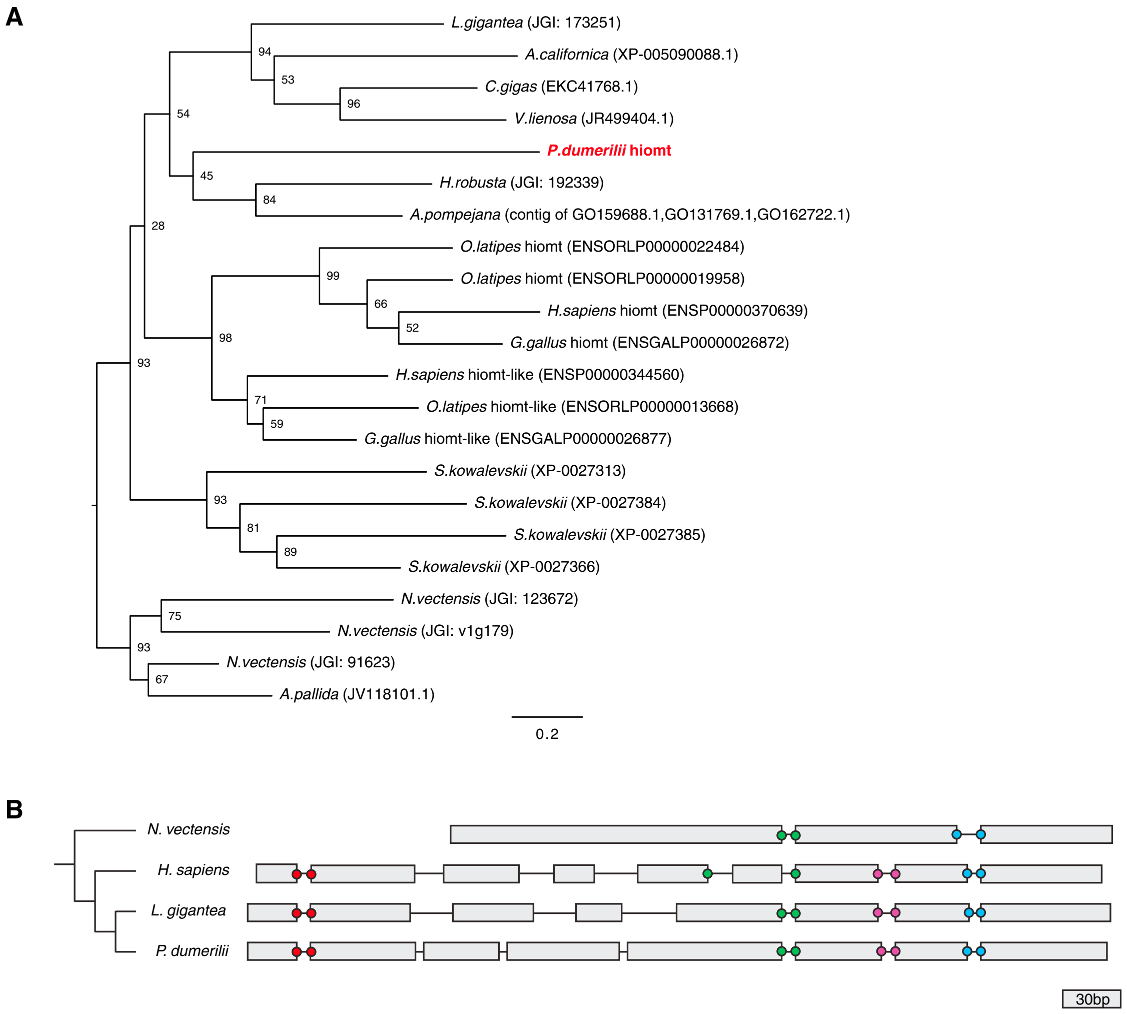

Figure S1. Phylogeny of *hiomt* Genes in Metazoa, Related to Figure 1

(A) Maximum likelihood phylogenetic tree of *hiomt* genes in Metazoa. *Hiomt* orthologs are present in Cnidaria (Anctil, 2009), in several annelids, in mollusks and in hemichordates. The accession numbers of the sequences used to build the tree (from NCBI, ENSEMBL, or JGI) are indicated in the figure. Numbers on the branches represent 100-times bootstrap support values.

(B) Genomic structure of representative *hiomt* genes. The gray rectangles represent exons, the lines between them introns (introns are not in scale). The positions of conserved splice sites are indicated by the colored dots.

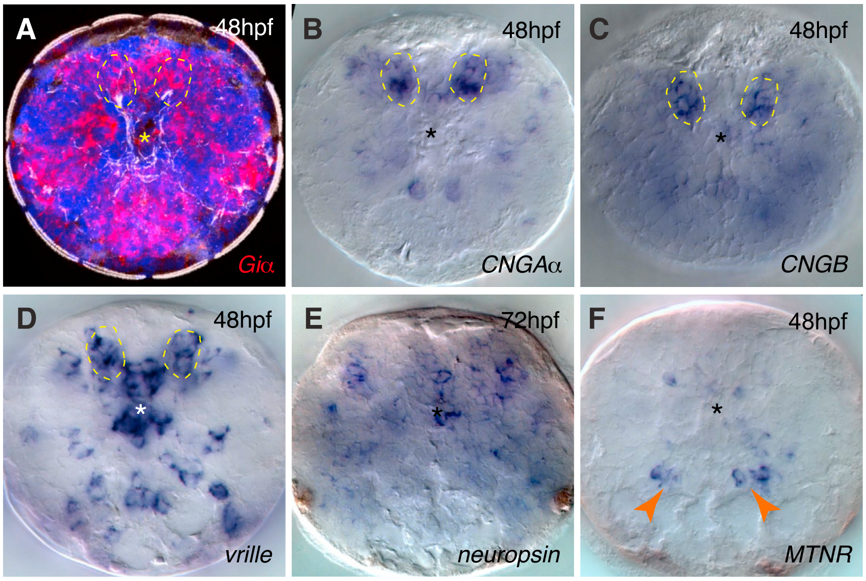

Figure S2. Additional Gene Expression Patterns in the *Platynereis* Dorsal Brain, Related to Figure 1

(A) Expression of the *Giα* subunit at 48hpf. Z-projection of a confocal stack, apical view. Red: gene expression; green: axonal scaffold; blue: DAPI.

(B–E) Gene expression in the extended ciliary photoreceptors region (dashed lines, apical views). Expression of the phototransduction cyclic-nucleotide gated channels *CNGAα* (B) and *CNGB* (C) at 48hpf; the circadian marker *vrille* at 48hpf (D) and *neuropsin* at 72hpf (E).

(F) Expression of *MTNR* in the developing brain at 48hpf. The arrowheads indicate the ventral cholinergic-*MTNR*+ neurons.

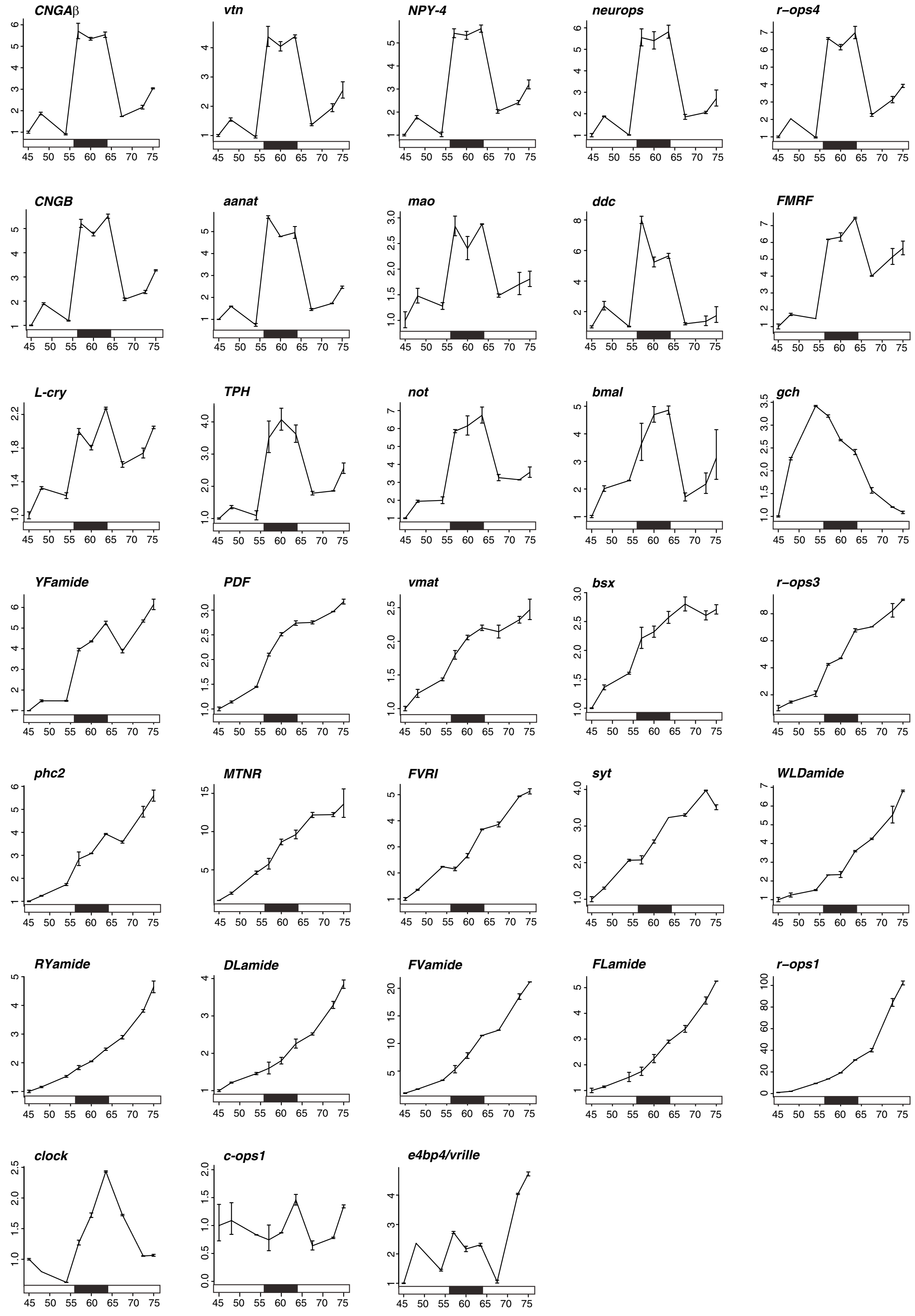

(legend on next page)

Figure S3. Gene Expression Dynamics during the Light-Dark Cycle, Related to Figure 2

The figure shows the gene expression profiles, relative to the first time point (45hpf), of genes assayed with the Fluidigm Dynamic Arrays. The same data set was used for clustering analysis in Figure 2. Here, gene expression relative to the first time point is plotted on the y axis, and developmental time (hpf = hours post fertilization) is plotted on the x axis. Each point represents the average of the two technical replicates (PreAmps), and error bars indicate standard deviation. The black bar below each plot indicates the night phase (from 56hpf to 64hpf).

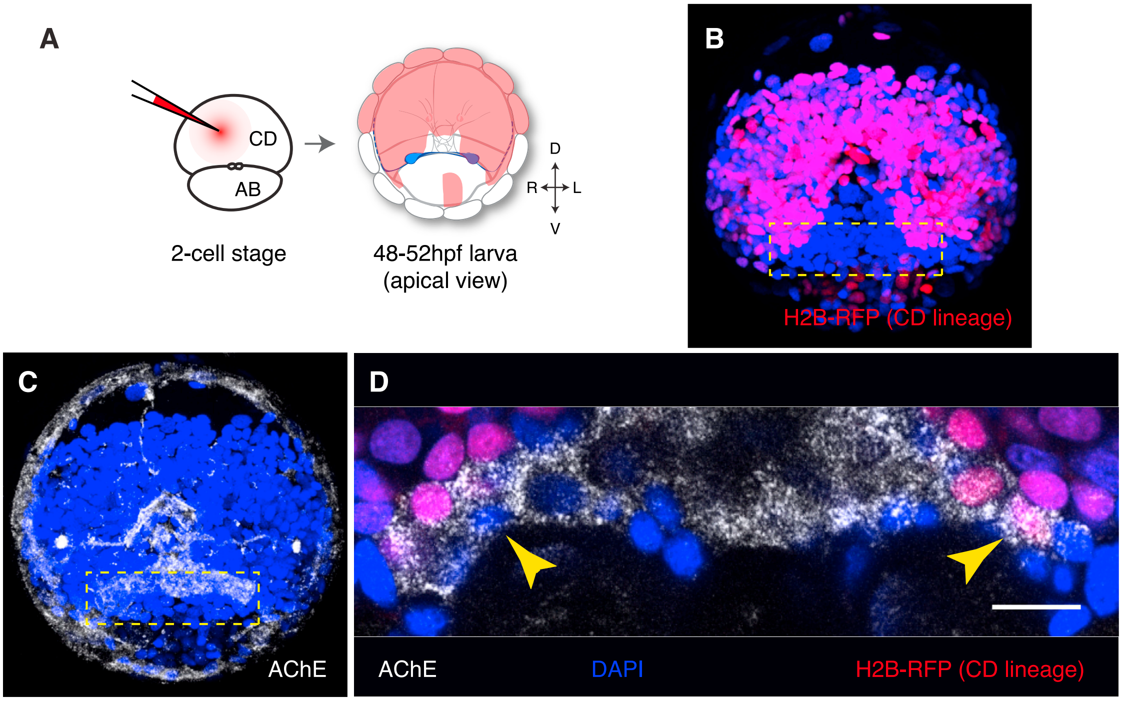

Figure S4. Position of the Ventral Cholinergic Neurons in Relationship to the Clonal Domains of the Larval Brain, Related to Figure 5

(A) Diagram showing the AB and CD blastomeres at 2-cell stage and the clonal domain of the episphere developing from the CD blastomere in 2-days old larvae (cfr. Ackermann et al., 2005 and Wilson, 1892).

(B) Distribution of H2B-RFP after injection of the CD blastomere with H2B-RFP mRNA, showing that the dorsal part of the larval brain develops from the CD blastomere (Z-projection of a confocal stack through the entire developing brain, apical view). Red: H2B-RFP, labeling the nuclei of the CD lineage; blue: DAPI.

(C) The same larva as in A, after acetylcholinesterase (AChE) staining (white, confocal reflection microscopy).

(D) Magnification of the larva in (B) and (C), showing the relationship of the AChE staining with the CD lineage. The arrowheads indicate two corresponding neurons, on the right and left side, which develop in stereotypic positions from AB and CD, respectively. Injection of the CD blastomere thus allows selective labeling of the neuron on the left side of the larva, but not of the corresponding one on the right. This same neuron is unique in its projection to the prototroch through the ventral cerebral commissure (compare Figure 5B and Lacalli, 1984). Scale bar = 10 μm.

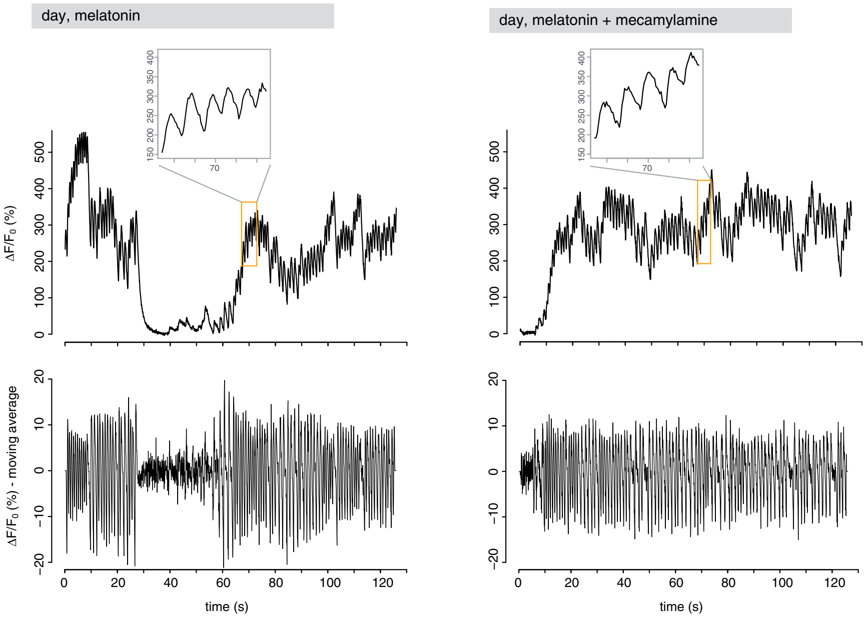

Figure S5. The Cholinergic Antagonist Mecamylamine Does Not Affect the Activity of Ventral Ciliomotor Neurons in Melatonin-Treated Larvae, Related to Figure 5

GCaMP6s fluorescence of a representative prototroch-projecting cholinergic neuron during daytime, after treatment with 100 μm melatonin (left) and subsequent addition of the acetylcholine receptor antagonist mecamylamine (100 μm, right). Upper row: GCaMP6s signal normalized with a global F_0; lower row: GCaMP6s signal normalized with a local F_0 (corresponding to the moving average over a window of 0.8 s), to highlight changes in fluorescence over a small time scale (see Experimental Procedures for details on the analysis).

Cell

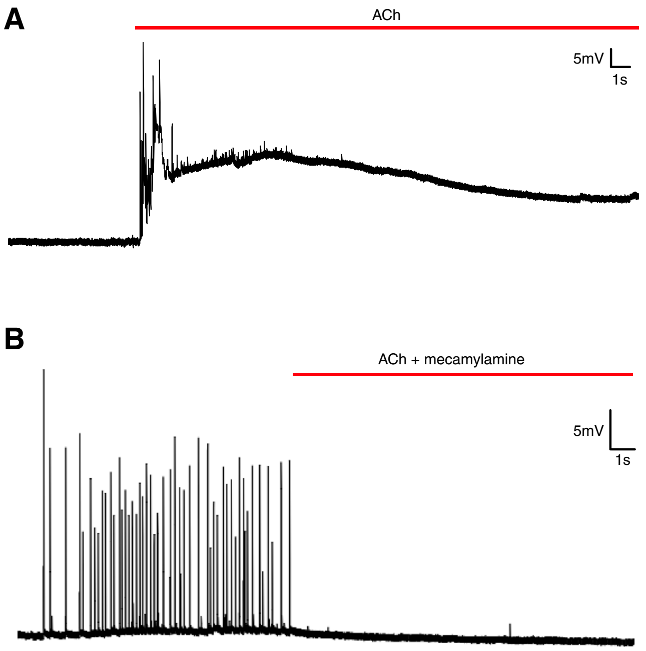

Figure S6. Effects of ACh and of the Cholinergic Antagonist Mecamylamine on the Prototroch Cells Electrical Activity, Related to Figure 6
(A) Representative intracellular recording from a prototroch cell where acetylcholine is applied (red line, ACh, 10mM) coincident with a phase of decreased activity. Acetylcholine elicits prototroch spiking and a depolarization of the postsynaptic prototroch cell.
(B) Representative intracellular recording from a prototroch cell after simultaneous application of acetylcholine and mecamylamine (red line, 10mM and 250 μM) during a phase of high activity. Mecamylamine suppresses synaptic activity and thereby the endogenous activity that would normally be evoked by acetylcholine.
